# Effects of Steam Explosion Combined with Ultrafine Grinding on the Physicochemical Properties, Antioxidant Capacity, and Volatile Profiles of *Morchella sextelata* Stipe Powders

**DOI:** 10.3390/foods15203748

**Published:** 2026-10-21

**Authors:** Jiaqi Jin, Fei Lao, Feng Kong, Lele Cao, Linhui Feng, Jia Chen, Jihong Wu, Xingfeng Guo

**Affiliations:** 1 Shandong Key Laboratory of Applied Technology for Protein and Peptide Drugs, School of Pharmaceutical Science and Food Engineering, Liaocheng University, Liaocheng 252000, China; jjq20190413@163.com (J.J.);; 2 College of Food Science & Nutritional Engineering, China Agricultural University, Beijing 100083, China

**Keywords:** mushroom by-products, steam explosion, particle size reduction, HS-SPME-GC-MS, volatile compounds

## Abstract

*Morchella sextelata* stipe is an underutilized by-product with potential for value-added food applications. This study investigated whether steam explosion (SE) pretreatment could enhance the effects of subsequent ultrafine grinding (UG) on the physicochemical properties, antioxidant capacity, and volatile profiles of stipe powders. Conventional grinding, UG, SE, and combined SE-UG treatments were compared using particle-size analysis, SEM, FT-IR, XRD, antioxidant assays, and HS-SPME-GC-MS. SE-UG reduced the median particle size (D50) from 88.88 to 10.46 μm and increased the water solubility index from 31.20% to 36.55%. It also caused extensive microstructural disruption and altered crystallinity without major changes in functional groups. SE-UG showed the highest DPPH and ABTS radical-scavenging activities, whereas SE and SE-UG exhibited similarly high total antioxidant capacity, with no significant difference between the two treatments. Volatile profiling showed treatment-dependent changes, with several furans and pyrazines occurring at higher relative abundances in SE-containing groups. These findings indicate that SE pretreatment facilitates subsequent UG and improves selected physicochemical and antioxidant properties while markedly modifying the volatile profile, supporting the value-added utilization of *M. sextelata* stipes.

## 1. Introduction

*Morchella sextelata*, an economically important edible mushroom species, has attracted increasing attention due to its nutritional value and potential health-promoting properties. The fruiting bodies of *M. sextelata* are rich in polysaccharides, dietary fiber, essential amino acids, phenolic compounds, and minerals, which contribute to various biological activities, including antioxidant and immunomodulatory effects [1]. With the rapid development of artificial cultivation technologies, *Morchella* production has expanded considerably, leading to increasing demands for efficient processing and comprehensive utilization of mushroom resources [2]. The commercial grading of dried morel products is largely determined by morphological characteristics, particularly stipe length, with high-grade products generally requiring partial or complete removal of the stipe to improve their appearance and market value. This practice inevitably generates considerable amounts of stipe by-products, which are often discarded or sold at low prices, resulting in resource waste and limiting the overall value enhancement of the morel industry [3]. Recent studies have further highlighted the importance of valorizing underutilized agricultural and food resources through nutritional characterization, functional ingredient development, and biotechnological conversion into value-added products [4,5,6,7]. In this context, appropriate processing and modification strategies may facilitate the effective utilization of these resources and promote their conversion into value-added food ingredients. Comparative studies on *M. sextelata* have demonstrated that the stipe possesses considerable nutritional potential, with no significant differences in crude polysaccharides, total protein, or total triterpenoid contents compared with the pileus. Moreover, the stipe contains higher levels of crude fiber, vitamins, total flavonoids, and ergothioneine, indicating its potential as a valuable functional ingredient [8].

Steam explosion (SE) is an environmentally friendly physical pretreatment technology that utilizes high-temperature and high-pressure saturated steam followed by instantaneous decompression [9]. The rapid pressure release generates mechanical shear forces through the explosive expansion of internal moisture, resulting in cell wall disruption, pore formation, and structural loosening of biomass matrices [10]. Previous studies have demonstrated that SE can effectively disrupt compact structural networks, improve pulverization performance, and enhance the physicochemical and functional properties of various food materials, including wheat bran, chicken bone, and *Ganoderma lucidum* powders [11,12,13]. Ultrafine grinding (UG) is another widely applied physical modification technique that reduces particle size through intensive mechanical forces, including impact, shear, and compression. The resulting increase in specific surface area can improve hydration properties, dissolution behavior, and the accessibility of bioactive components [14]. However, the effectiveness of UG is strongly influenced by the structural characteristics of the raw material, and its ability to disrupt highly compact biological matrices may be limited [15]. Therefore, combining SE pretreatment with UG may provide a complementary processing strategy, in which SE facilitates structural disruption and loosening of the cellular matrix, while UG further promotes particle size reduction and surface exposure.

Steam explosion has also been shown to improve the grindability and physicochemical properties of mushroom-derived materials. For example, Kong et al. [13] reported that steam explosion of *Ganoderma lucidum* significantly disrupted its compact structure and increased the cell-wall breakage rate by 129.51%, resulting in a 64.87% reduction in average particle size and an 8.95% decrease in grinding power consumption. Steam explosion also increased the water solubility index by 79.27%, indicating substantial modification of the structural and hydration characteristics of the material [16,17]. These findings suggest that steam explosion can effectively weaken the structural resistance of mushroom tissues to size reduction and may therefore serve as a useful pretreatment before subsequent grinding. However, whether steam explosion can similarly facilitate subsequent ultrafine grinding of *M. sextelata* stipes remains unclear. This question is particularly relevant to *M. sextelata* stipes because their compact cellular structure may limit the effectiveness of mechanical size reduction alone. Thus, the key question addressed in this study was whether SE-induced structural disruption could facilitate subsequent UG and lead to physicochemical and functional changes beyond those obtained by UG alone. We hypothesized that SE pretreatment would increase the susceptibility of the stipe matrix to subsequent ultrafine grinding, resulting in greater particle-size reduction and surface exposure than UG alone. We further expected that these structural changes would be reflected in the physicochemical and functional properties of the powders, while the high-temperature SE process would have a greater influence on volatile profile transformation [18].

Volatile profiles were also examined because the two treatments are expected to affect aroma compounds through different mechanisms. The high temperature and rapid pressure release during SE may promote the formation or transformation of thermally derived volatile compounds [19], whereas UG mainly reduces particle size and increases surface exposure, which may affect the release of existing volatile compounds [20]. For *M. sextelata* stipe powder, changes in characteristic mushroom-associated compounds and process-derived volatiles can therefore help distinguish treatment-related changes associated with thermal-pressure processing from those associated with mechanical size reduction. In the present study, volatile analysis was intended to characterize treatment-associated changes in headspace composition rather than to define sensory improvement. No sensory evaluation or odor activity value analysis was performed. Accordingly, this study aimed to systematically investigate the effects of conventional grinding (CG), ultrafine grinding (UG), steam explosion (SE), and sequential steam explosion followed by ultrafine grinding (SE-UG) on the physicochemical, structural, antioxidant, thermal, and volatile characteristics of *M. sextelata* stipe powders. Specifically, we aimed to (i) characterize the changes in particle size, morphology, physicochemical properties, structural features, and thermal stability induced by the different treatments; (ii) evaluate their effects on antioxidant capacity; and (iii) characterize treatment-associated changes in volatile profiles. Particular attention was given to determining whether SE pretreatment could facilitate subsequent ultrafine grinding and whether the sequential treatment produced distinct or complementary effects compared with the individual treatments.

## 2. Materials and Methods

### 2.1. Materials

The fruiting bodies of *M. sextelata* were purchased from a local agricultural product market in Liaocheng, Shandong, China, on 20 March 2026, with a total fresh weight of approximately 20 kg. The samples were obtained as a single batch, cleaned to remove impurities, and dried in an oven at a constant temperature of 25 °C until a constant weight was achieved. The dried samples were subsequently stored in sealed bags for further analysis. The samples were obtained as a single batch and subjected to the same pretreatment. For each analytical determination, three measurements were performed as analytical replicates. The DPPH radical scavenging activity assay kit and ABTS radical scavenging activity assay kit were purchased from Solarbio Science & Technology Co., Ltd. (Beijing, China). Other chemicals and reagents were obtained from Shanghai Macklin Biochemical Co., Ltd. and Aladdin Biochemical Technology Co., Ltd. (Shanghai, China). All chemicals and solvents used in this study were of analytical grade.

### 2.2. Nutritional Value of the Stipe and Pileus in M. sextelata (Dry Weight Basis)

#### 2.2.1. Determination of Fat

The fat content of the *M. sextelata* stipes and pileus was determined using a Soxhlet extraction apparatus, according to the method described by Sajad [21] with slight modifications. Briefly, the stipe samples were extracted using a SOX406 fat analyzer (Hanon Advanced Technology Group Co., Ltd., Jinan, Shandong, China). The continuous reflux extraction was conducted at 55 °C for 6 h. Following solvent recovery, the receiving flask containing the extracted fat was dried in an oven at 105 °C to a constant weight. The crude fat content was calculated using the following Equation (1):

(1) $$\mathrm{Fat}\;(\%)\;=\;\frac{m_{2}\;-{\;m}_{1}}{m_{0}}\;\times \;100$$

where m_2_ is the total weight of the receiving flask and the extracted fat (g), m_1_ is the weight of the empty flask (g), and m_0_ is the weight of the sample (g).

#### 2.2.2. Determination of Crude Protein

The crude protein content of the lyophilized *M. sextelata* stipe powder was determined using the Kjeldahl method. Briefly, 0.500 g of the lyophilized stipe powder was mixed with CuSO_4_, K_2_SO_4_, and H_2_SO_4_ in a digestion tube. The mixture was digested in a heating block at 420 °C for 1 h until a clear, blue-green solution was obtained. After cooling, the digestate was subjected to automatic distillation and titration using a K9840 semi-automatic Kjeldahl analyzer (Hanon Advanced Technology Group Co., Ltd., China). The crude protein content was calculated using the following Equation (2):

(2) $$\mathrm{Crude}\;\mathrm{protein}\;(\%)\;=\;\frac{{(V}_{1}\;-{\;V}_{0})\;\times \;C\;\times \;0.014}{m}\;\times \;6.25\;\times \;100\;$$

where V_1_ is the volume of the standard acid solution consumed by the sample (mL), V_0_ is the volume of the standard acid solution consumed by the blank (mL), C is the molar concentration of the standard acid solution (mol/L), m is the mass of the lyophilized stipe sample (g).

#### 2.2.3. Determination of Crude Fiber

The crude fiber content of the lyophilized *M. sextelata* stipe powder was determined using an F800 fiber analyzer (Hanon Advanced Technology Group Co., Ltd., China). Briefly, an accurately weighed amount of the lyophilized stipe sample was placed into a specialized filtering crucible. The sample was subjected to sequential digestion with boiling dilute acid and dilute alkali solutions in the analyzer. Following digestion, the residue was washed repeatedly with hot water until neutral. The crucible containing the digested residue was dried in an oven to a constant weight. Subsequently, the dried residue was incinerated in a muffle furnace and weighed again to determine the ash content. The crude fiber was calculated using the following Equation (3):

(3) $$\mathrm{Crude}\;\mathrm{fiber}\;(\%)\;=\;\frac{m_{1}\;-{\;m}_{2}}{m_{0}}\;\times \;100\;$$

where m_1_ is the total weight of the crucible and the dried residue after digestion (g), m_2_ is the total weight of the crucible and the ash after incineration (g), and m_0_ is the initial weight of the lyophilized stipe sample (g).

### 2.3. Preparation of Morchella sextelata Stipe Powder

The fresh *M. sextelata* stipes were dried under cold air at 25 °C. The CG group was prepared by milling and passing the material through a 40-mesh sieve. For the UG group, the samples were coarsely ground and then subjected to ultrafine grinding (JWM-F, Jingjiang Huiheng Machinery Manufacturing Co., Ltd., Taizhou, China) for 10 min. For steam explosion treatment, the dried stipes were cut into approximately 1 cm pieces, and their moisture content was adjusted to 20% by adding distilled water as an integral moisture-conditioning step for the steam explosion process. The moistened samples were then treated using a dedicated steam explosion apparatus (QBS-80, Qingzheng Eco-Technology (Suzhou) Co., Ltd., Suzhou, China) at 0.8 MPa for 90 s, following a previously reported protocol [22]. This condition was adopted as a practical processing condition rather than an optimized condition specifically established for *M. sextelata*. The materials were then rapidly decompressed into a receiving device to terminate the treatment. The steam-exploded materials were immediately freeze-dried to minimize additional thermal effects during post-treatment drying and to preserve, as far as possible, the structural and physicochemical changes induced by steam explosion. After freeze-drying, the materials were divided into two portions. One portion was conventionally ground and passed through a 40-mesh sieve, designated as the SE group, whereas the other portion was subjected to ultrafine grinding for 10 min, designated as the SE-UG group.

### 2.4. Morphological Analysis of Morchella sextelata Stipes

Scanning electron microscopy (SEM) has been widely used to characterize the microstructural and morphological changes in food powders subjected to steam explosion and superfine grinding [23]. The powder samples were uniformly mounted on copper stubs using double-sided conductive carbon tape, and excess powder was removed. Then, the samples were sputter-coated with gold (5 mA, 100 s). The scanning electron microscope (SEM) (Thermo Fisher Scientific, Waltham, MA, USA) was used to obtain SEM images of *M. sextelata* stipe powders at a voltage of 10 kV [24]. Images were captured using an ETD detector in secondary electron (SE) mode at magnifications of 2500× and 10,000×.

### 2.5. Particle Size Distribution of Morchella sextelata Stipes

Particle size distribution of *M. sextelata* stipe powders was measured using a laser particle size instrument (NKT3100-H, Shandong NKT Analytical Instrument Co., Ltd., China) at room temperature. The measurement was conducted in a wet dispersion mode with deionized water as the dispersion medium. The system parameters were set as follows: the refractive index of the medium was 1.33, the refractive index of the sample was 1.61, and the absorption was 0.1. The sample concentration was adjusted to maintain an obscuration rate of approximately 15%. The recorded parameters included D_10_, D_50_, D_90_, surface-weighted mean diameter (D_[3,2]_), volume-weighted mean diameter (D_[4,3]_), and specific surface area (S/V).

### 2.6. Color Measurement of Morchella sextelata Stipe Powder

The color parameters of the modified *M. sextelata* stipe powders were analyzed using a spectrophotometer (GYJ-S110, Shenzhen GOYOJO Industrial Technology Co., Ltd., Shenzhen, China), following the method described by Xing et al. [25] with minor modifications. The powder samples were placed in transparent and flat-bottomed plastic Petri dishes to completely cover the bottom. Prior to measurement, the instrument was calibrated using the provided standard black and white calibration plates. L* (lightness), a* (color range from green to red), and b* (color range from blue to yellow) values, and Δ*E* were recorded and analyzed.

### 2.7. Determination of Water-Holding Capacity (WHC), Oil-Holding Capacity (OHC), and Water Solubility Index (WSI)

The WHC and OHC were determined according to the method previously described [26] with minor modifications. For WHC, 1.000 g of the sample was suspended in deionized water at a solid-to-liquid ratio of 1:20 (g/mL) and stirred. Then, the suspension was incubated in a water bath at 60 °C for 40 min. The mixture was centrifuged at 5000 r/min for 20 min at room temperature. The supernatant was discarded, and the residue was weighed.

For OHC, 1.000 g of the sample was mixed with food-grade oil (1:8, g/mL). After vortexing, the mixture was incubated in a water bath at 37 °C for 1 h. Following centrifugation under the same conditions, the free oil was decanted, and the residue was weighed.

WHC and OHC were calculated using the following Equation (4). The final results were calculated on a dry basis. The variables in the formula are defined as follows:

(4) $$\mathrm{WHC}/\mathrm{OHC}\;(g/g)\;=\;\frac{m_{2}-\;m_{1\;}-{\;m}_{0}\;\times \;\left(1-\omega \right)}{m_{0}\;\times \;\left(1-\omega \right)}$$

where m_0_ is the actual mass of the sample (g); ω is the initial moisture mass fraction (%); m_1_ is the mass of the empty centrifuge tube (g); and m_2_ is the total mass of the centrifuge tube and the precipitate (g).

The WSI was measured by the modified method as previously described [27]. A 1.25 g powder sample with 15 mL of distilled water was vigorously vortexed to ensure complete dispersion and incubated in a 30 °C water bath for 30 min. The mixture was centrifuged at 3000× *g* for 10 min; the supernatant was dried at 105 °C to a constant weight. The WSI was calculated as follows (5):

(5) $$\mathrm{WSI}\;(\%)\;=\;\frac{m_{2\;}-\;m_{1}}{m\;\times \;\left(1-\omega \right)}$$

where m_2_ is the mass of the glass bottle containing dried soluble solids (g); m_1_ is the mass of the empty glass bottle (g); and m is the dry mass of the sample (g); ω is the initial moisture mass fraction (%).

### 2.8. Infrared Spectrum Information of Morchella sextelata Stipes

The modified powders were mixed with spectroscopic-grade KBr at a mass ratio of 1:200 and pressed into pellets. The FT-IR spectra were then recorded using a Fourier transform infrared spectrometer (FTIR-850, Tianjin Gangdong Sci. & Tech. Co., Ltd., Tianjin, China) over a wavenumber range of 4000–400 cm^−1^ with a resolution of 4 cm^−1^.

### 2.9. X-Ray Diffraction (XRD) Analysis

The crystalline structure of the *M. sextelata* stipe powders was analyzed using an X-ray diffractometer (DX-2700B, Dandong Haoyuan Instrument Co., Ltd., Dandong, China) operating at a voltage of 40 kV and a current of 40 mA. The samples were scanned over a 2θ diffraction angle ranging from 5° to 50° with a step size of 0.02°.

### 2.10. Differential Thermogravimetric Profiles of Morchella sextelata Stipe Powder

The thermogravimetric (TG) and differential thermogravimetric (DTG) profiles of the dry *M. sextelata* stipe powder were measured using a vacuum-tight synchronous TGA-DSC thermal analyzer (STA 449 F5, NETZSCH Gerätebau GmbH, Selb, Germany). The measurement was conducted under a high-purity nitrogen atmosphere with a flow rate of 50 mL/min, heating from 30 °C to 600 °C at a rate of 10 °C/min.

### 2.11. Evaluation of Antioxidant Capacities

The antioxidant capacities of *M. sextelata* stipe powders subjected to different physical modifications were evaluated using 2,2-diphenyl-1-picrylhydrazyl (DPPH) radical scavenging, 2,2′-azino-bis (3-ethylbenzothiazoline-6-sulfonic acid) (ABTS) radical scavenging, and total antioxidant capacity (T-AOC) assays with commercial assay kits (Solarbio Science & Technology Co., Ltd., Beijing, China). For sample extraction, 0.4 g of each powder sample was accurately weighed and extracted with 10 mL of deionized water, corresponding to a solid-to-liquid ratio of 1:25 (g/mL). The mixture was thoroughly shaken and then centrifuged at 10,000 rpm for 10 min at 4 °C. The resulting supernatant was collected and used as the sample extract for subsequent antioxidant assays. Based on preliminary experiments, different concentrations of sample extracts were prepared for the DPPH and ABTS assays, with concentration ranges of 0–25 mg/mL and 0–30 mg/mL, respectively. For the T-AOC assay, the sample extract concentration was set at 40 mg/mL. All assays were conducted according to the manufacturer’s instructions. The absorbance values were measured at 515 nm, 405 nm, and 593 nm for the DPPH, ABTS, and T-AOC assays, respectively. Each sample was analyzed in triplicate. The DPPH and ABTS radical scavenging activities were calculated using the following Equation (6):

(6) $$\frac{\mathrm{ABTS}}{\mathrm{DPPH}}\mathrm{radical scavenging rate}\left(\%\right)=\frac{A_{\mathrm{blank}}-\left(A_{\mathrm{test}}-{\;A}_{\mathrm{control}}\right)}{A_{\mathrm{blank}}}\times 100$$

The T-AOC values were calculated according to the calibration curve provided by the kit and expressed as μmol Fe^2+^/g dry weight (DW).

### 2.12. Headspace Solid-Phase Microextraction Coupled with Gas Chromatography-Mass Spectrometry (HS-SPME-GC-MS) Analysis of Volatile Compounds in Morchella sextelata Stipe Powders

Volatile compounds in *M. sextelata* stipe samples were analyzed using HS-SPME-GC-MS according to a previously reported method with minor modifications [28]. Briefly, 1.0 g of each sample was transferred into a 20 mL headspace vial, followed by the addition of 3 mL of saturated NaCl solution and 10 μL of 2-methyl-3-heptanone solution (100 mg/L) as the internal standard. The vial was immediately sealed and equilibrated in a thermostatic shaker at 55 °C and 450 rpm for 20 min. Subsequently, volatile compounds were extracted using a DVB/CAR/PDMS fiber (Supelco, Bellefonte, PA, USA) for 40 min under the same conditions. After extraction, the fiber was inserted into the GC injector and thermally desorbed for 180 s.

GC-MS analysis was performed using a gas chromatography-mass spectrometry system (QP2020-NX, Shimadzu Corporation, Kyoto, Japan) equipped with a DB-WAX capillary column (30 m × 0.25 mm i.d. × 0.5 μm film thickness, Agilent Technologies, Santa Clara, CA, USA). Helium (99.999% purity) was used as the carrier gas at a constant flow rate of 1.0 mL/min. The oven temperature program was set as follows: an initial temperature of 60 °C for 2 min, increased to 170 °C at 2 °C/min, followed by an increase to 240 °C at 5 °C/min, and maintained for 4 min. The mass spectrometer was operated in electron ionization (EI) mode at 70 eV, with the ion source temperature set at 220 °C. Volatile compounds were tentatively identified by comparing their mass spectra with those in the NIST spectral library. The relative abundance of each volatile compound was semi-quantitatively estimated using 2-methyl-3-heptanone as the internal standard based on the peak-area ratio. The resulting values were interpreted as relative abundances rather than absolute concentrations. Each sample was analyzed in triplicate.

### 2.13. Statistical Analysis

All experiments were repeated three times, and the data were statistically analyzed through one-way analysis of variance (ANOVA). A very significant difference was defined as *p* < 0.01, and a significant difference as *p* < 0.05 through Duncan’s multiple tests. Statistical analysis was mainly conducted using IBM SPSS 29.0.2.0 software.

For multivariate analysis of volatile compounds, the relative abundance data were analyzed using R software (version 4.5.2) in RStudio. Partial least squares discriminant analysis (PLS-DA) was performed using the mixOmics package as an exploratory supervised classification approach to visualize treatment-associated separation. Seven-fold cross-validation with 10 repeats and a 200-times permutation test were used to assess model performance and the likelihood of obtaining comparable separation after random class assignment. Variable importance in projection (VIP) scores were calculated to rank the contribution of individual volatile compounds to group discrimination; because of the small sample size, VIP scores were interpreted as exploratory variable-ranking measures rather than as validated biomarkers. For hierarchical cluster analysis (HCA), the relative abundance data were Z-score standardized. HCA was performed based on Euclidean distance using the complete linkage method, and the clustering results were visualized as a heatmap using the ComplexHeatmap package (version 2.28.0).

## 3. Results and Discussion

### 3.1. Nutritional Composition of the Pileus and Stipe of Morchella sextelata

**Figure 1:**
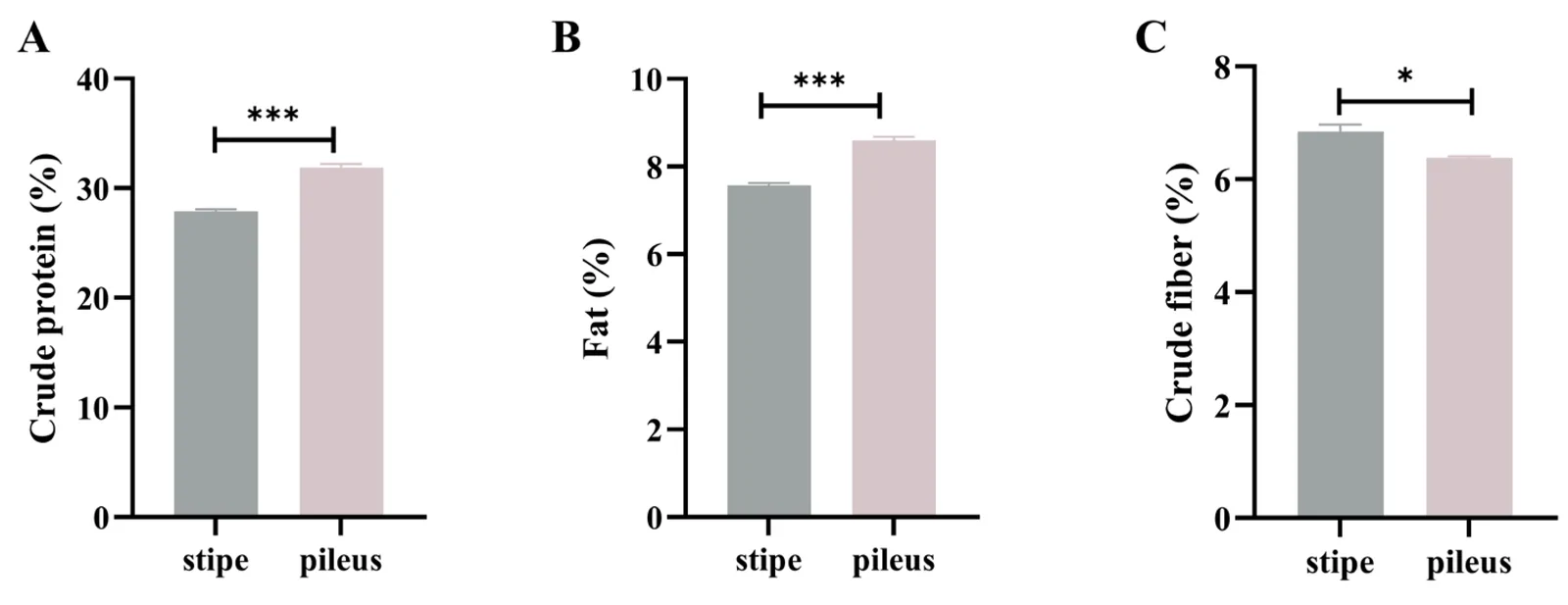
Proximate composition of *M. sextelata* stipe and pileus. (**A**) Crude protein; (**B**) Crude fat; (**C**) Crude fiber. Data are presented as mean ± SD (*n* = 3). Asterisks indicate significant differences between stipe and pileus (^*^p < 0.05; ^***^p < 0.001).

The nutritional composition of the pileus and stipe of *M. sextelata* was compared by determining their crude protein, crude fat, and crude fiber contents (Figure 1). Distinct compositional differences were observed between the two morphological parts. Specifically, the pileus contained significantly higher levels of crude protein (31.88%) and crude fat (8.59%) than the stipe (*p* < 0.001). This difference may be associated with the distinct structural and physiological characteristics of the two parts of the fruiting body. In contrast, the stipe exhibited a significantly higher crude fiber content (6.85%, *p* < 0.05). Previous comparative analysis of *M. sextelata* also reported significant differences in dietary fiber composition between the pileus and stipe, further highlighting the nutritional value of the stipe as a potential source of fiber [8].

Similar variations in proximate composition have also been reported among other *Morchella* species. For example, *Morchella importuna* fruitbodies cultivated under a rice–pea–*M. importuna* rotation system contained 37.32 g/100 g crude protein, 4.04 g/100 g crude fat, and 10.06 g/100 g crude fiber on a dry-weight basis [29]. More recently, comparative analysis of four *Morchella* species (*M. eximia*, *M. importuna*, *M. tridentina*, and *M. andinensis*) showed that their crude protein contents ranged from 31.08% to 36.61% of dry matter, while crude fiber contents ranged from 15.27% to 18.50% and ether extract contents ranged from 3.80% to 6.06% [30]. These findings indicate that morel fruitbodies generally contain appreciable levels of protein and structural fiber, although their nutritional composition may vary among species and growing environments.

The relatively higher crude fiber content observed in the stipe in the present study suggests a greater contribution of structural components to its matrix, which may influence its physicochemical properties and processing characteristics. Despite its lower protein and fat contents compared with the pileus, the stipe therefore remains a promising fiber-rich raw material for further processing and value-added utilization. This structural characteristic also provides a basis for investigating modification strategies to improve its processing characteristics and utilization efficiency.

### 3.2. Scanning Electron Microscopy (SEM) Observation

**Figure 2:**
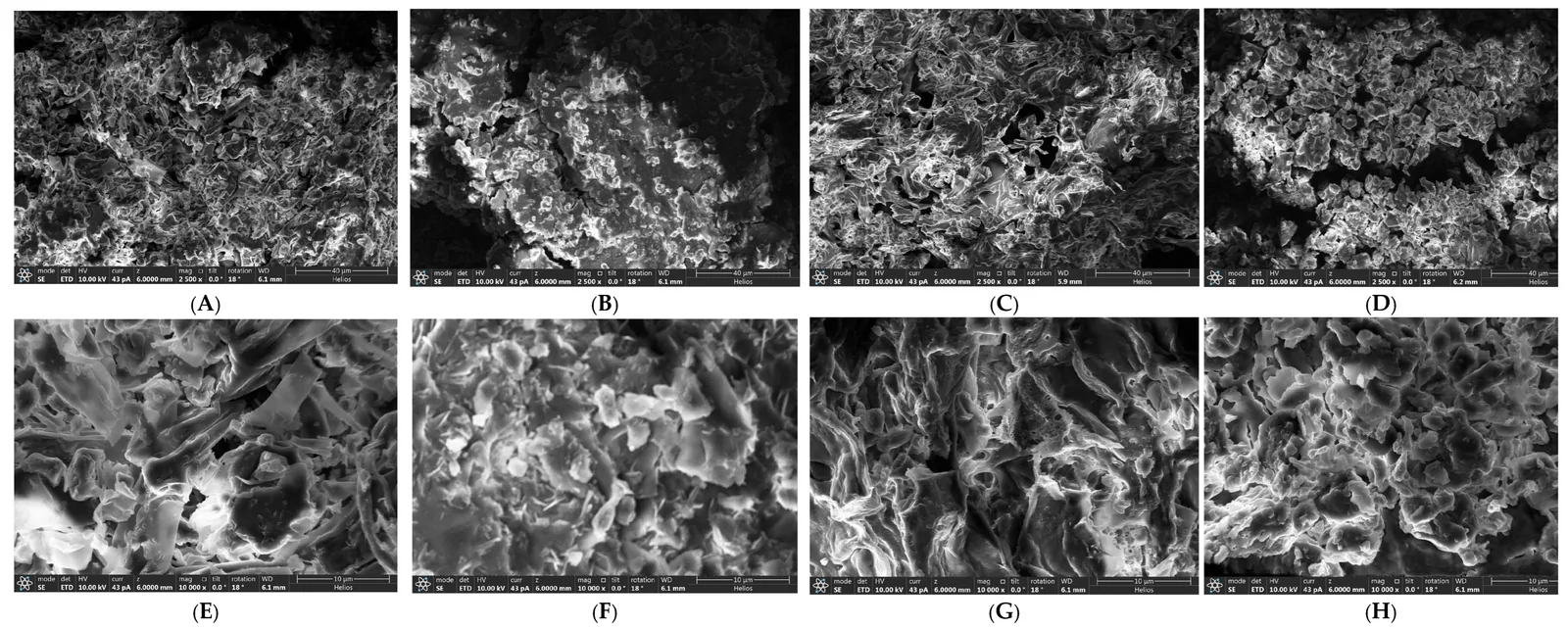
Scanning electron microscopy images of *M. sextelata* stipe powders subjected to different physical modification treatments. (**A**,**E**) CG; (**B**,**F**) UG; (**C**,**G**) SE; (**D**,**H**) SE-UG. Images (**A**–**D**) were obtained at 2500× magnification, while images (**E**–**H**) were obtained at 10,000× magnification.

SEM images of *M. sextelata* stipe powders subjected to different physical modification treatments are shown in Figure 2. As shown in Figure 2A,E, the CG group exhibited a dense, coarse, and relatively intact block-like structure with limited exposure of the internal microstructure Figure 2B,F showed that the original dense structures were fragmented by mechanical shear forces into smaller angular particles with irregular protrusions. The increased surface roughness may contribute to an increased surface area available for interactions with water molecules, and these morphological changes are consistent with previous studies [31,32].

In contrast, the SE group (Figure 2C,G) exhibited a loose, reticular, and porous structure. This phenomenon might be attributed to the penetration of high-pressure steam and the instantaneous pressure release during steam explosion [25]. For the SE-UG group (Figure 2D,H), the intact porous architecture largely disappeared and was replaced by extremely fine, loose, and relatively uniform agglomerates of irregular particles, resulting in a substantially increased specific surface area. These microstructural characteristics indicate that sequential SE followed by UG produced extensive disruption of the tissue network and deep fragmentation, providing a structural basis that may facilitate solvent access to internal components [33].

### 3.3. Particle Characteristics of Morchella sextelata Stipe Powder

**Figure 3:**
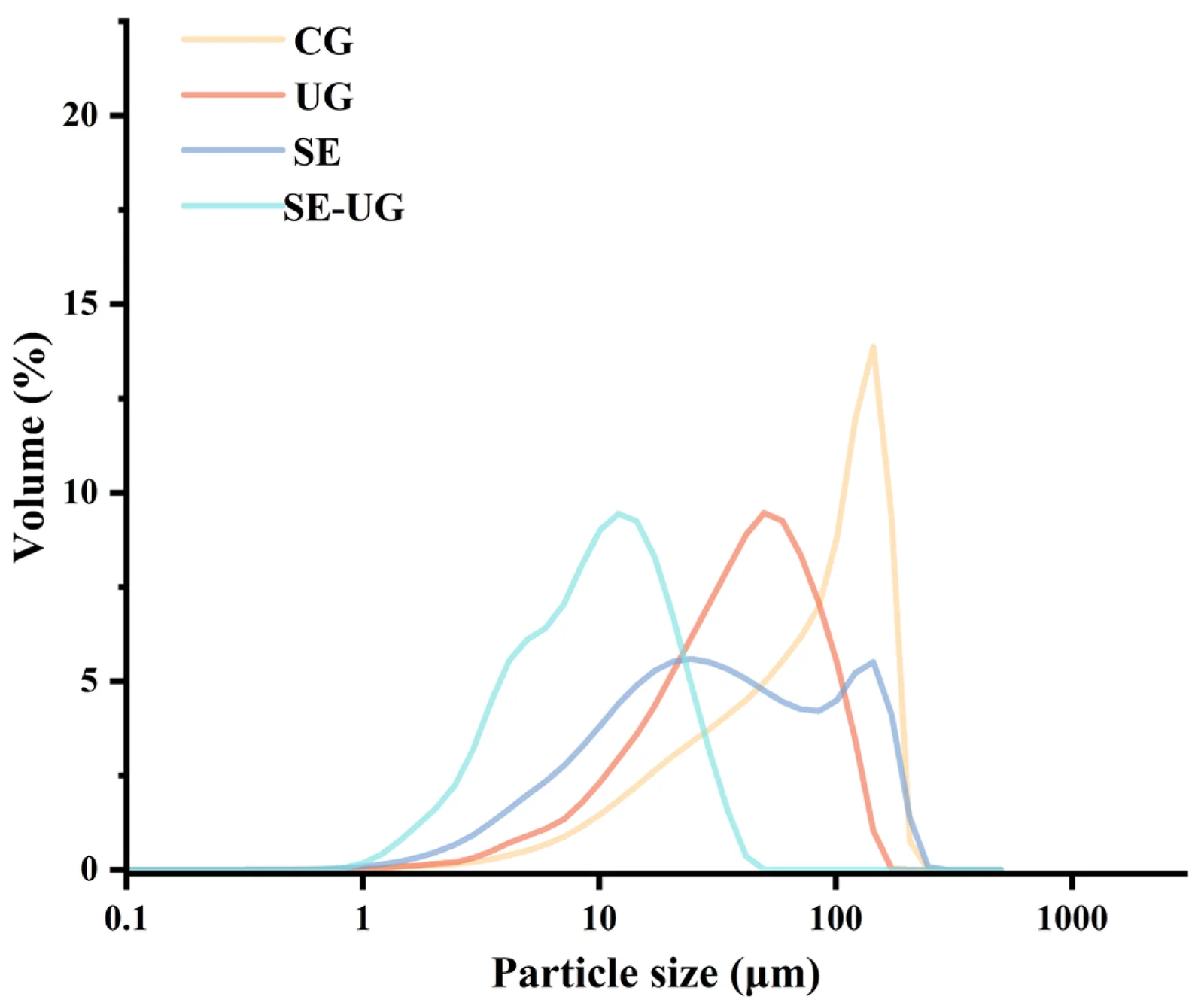
Particle size distribution curves of *M. sextelata* stipe powders subjected to different physical modification treatments.

The particle size distributions of various *M. sextelata* stipe powders are shown in Figure 3. The CG group exhibited a typical narrow unimodal distribution, with particles primarily concentrated in the coarse range (>100 μm). Following ultrafine grinding, the main peak of the UG group shifted toward smaller particle sizes, which could be attributed to fragmentation of the structural matrix by intensive mechanical forces. In contrast, the SE group displayed a bimodal distribution with a decreased peak height. A similar heterogeneous distribution after steam explosion has been reported previously. This phenomenon may be associated with the disruption and modification of the structural matrix during the treatment, which can alter the size and dispersion characteristics of the resulting particles [13]. However, the combined SE-UG treatment effectively eliminated this agglomeration. The distribution curve of SE-UG further shifted leftward and formed a sharp peak around 10 μm.

The particle characteristics of various *M. sextelata* stipe powders are summarized in Table 1. The CG group possessed the largest particle size, with D_50_ and D_[4,3]_ reaching 88.88 μm and 92.16 μm, respectively. The UG treatment significantly reduced the D_50_ to 44.39 μm (*p* < 0.05). For the SE group, although the D_50_ decreased to 33.87 μm, the D_90_ reached as high as 148.94 μm, and the span increased sharply to 4.198, indicating a deterioration in powder uniformity [34].

The combined SE-UG modification achieved the lowest values for all particle size indices. Specifically, the D_50_ and D_90_ significantly decreased to 10.46 μm and 24.47 μm, respectively, with the span dropping to 1.968. Meanwhile, the increase in the proportion of fine particles led to a reduction in D_[3,2]_ to 7.27 μm, resulting in a substantial increase in the specific surface area (S/V) to 8256.48 cm^2^/cm^3^, which was approximately 4.85 times that of the CG group. These results demonstrate that the combined process successfully broke the physical cross-linking of particles and greatly increased the effective contact area of the powder; smaller particle size was considered to be more favorable for bioaccessibility and bioavailability [35].

### 3.4. Changes in Surface Color of Morchella sextelata Stipes Powder

Appearance is an important factor for consumers when choosing produce, and color is a major factor in determining appearance [36]. As shown in Table 2, the different physical modification treatments significantly affected the color parameters of *M. sextelata* stipe powders (*p* < 0.05). The L* value represents lightness, ranging from black (0) to white (100), whereas positive and negative a* values indicate redness and greenness, respectively, and positive and negative b* values indicate yellowness and blueness, respectively [37]. Compared with the CG group, SE markedly decreased the L* value from 80.41 ± 0.29 to 68.76 ± 0.25, while increasing the a* and b* values. Meanwhile, the total color difference (Δ*E*) reached 11.70, indicating a pronounced change in surface color. The darker coloration observed after SE may be associated with non-enzymatic browning reactions, including Maillard reactions and caramelization, under the thermal conditions of steam explosion [38].

Compared with the SE group, the SE-UG group exhibited a significantly higher L* value of 75.13 ± 0.12, while the Δ*E* value decreased to 5.03, indicating that subsequent ultrafine grinding partially moderated the color changes associated with SE treatment. This change may be related to the substantial reduction in particle size and the accompanying increase in specific surface area after ultrafine grinding, as changes in particle size can influence the diffuse reflection and scattering of incident light [39,40]. Overall, these results indicate that subsequent UG partially reduced the extent of SE-associated color alteration in *M. sextelata* stipe powder.

### 3.5. Effects of Combined Modification on Hydration Properties

**Figure 4:**
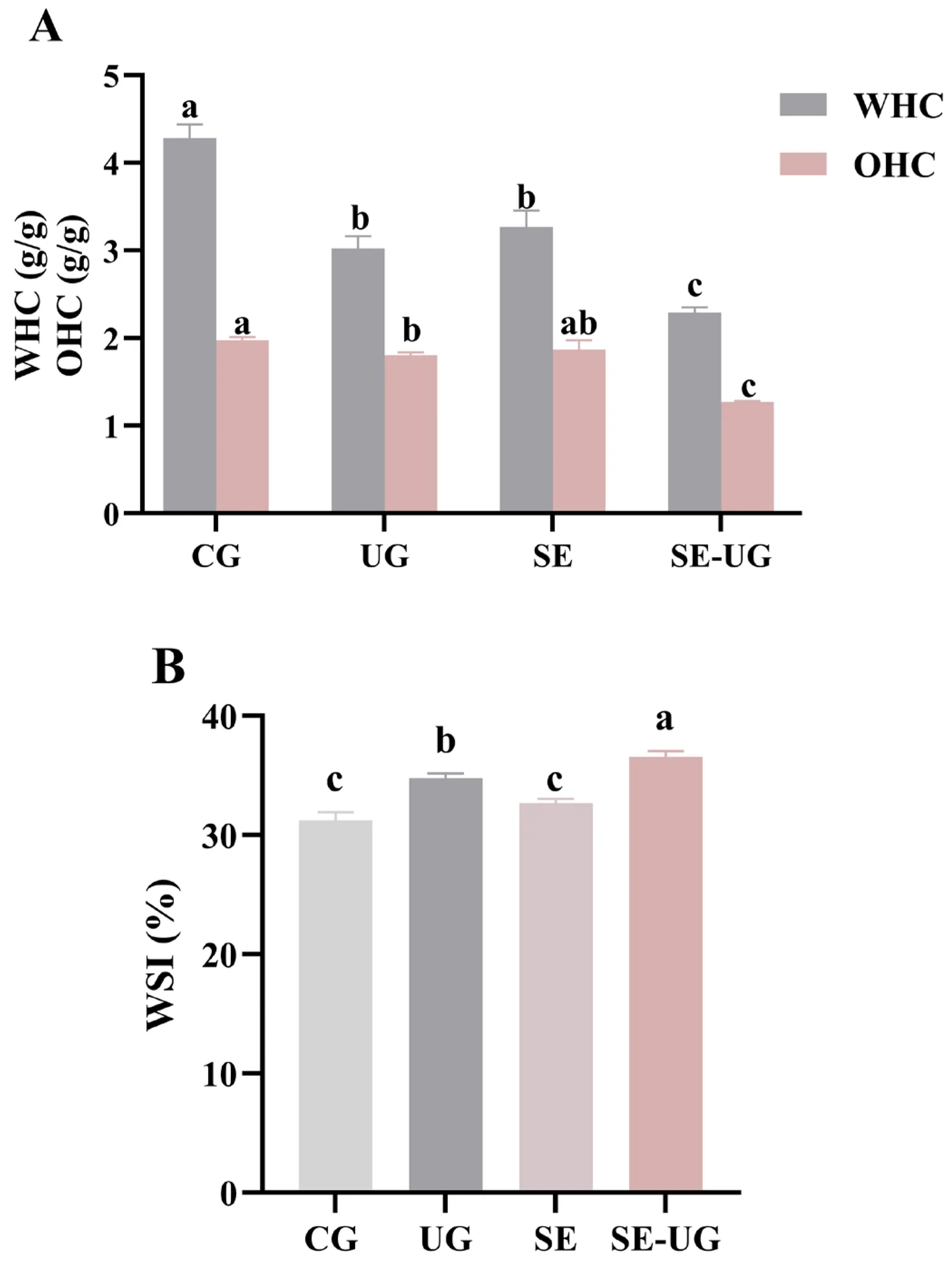
Effects of different physical modification treatments on the hydration properties of *M. sextelata* stipe powders. (**A**) WHC and OHC. (**B**) WSI. Different lowercase letters indicate significant differences (*p* < 0.05).

WHC and OHC are critical indicators for evaluating the spatial structure and potential gastrointestinal functions [41]. As depicted in Figure 4 the modification treatments involving UG, SE, and their combination significantly decreased the WHC and OHC of *M. sextelata* stipe powders (*p* < 0.05). The WHC values decreased from 4.28 g/g in the CG group to 3.02, 3.27, and 2.28 g/g in the UG, SE, and SE-UG groups, respectively, while the OHC exhibited a similar decreasing trend. The SE-UG group exhibited the lowest retention capacities among all samples. The reduction in hydration properties within the UG group is closely related to the applied mechanical shear forces. Ultrafine grinding reduces particle size and increases the specific surface area of the powders. However, this mechanical treatment destroys the original internal pore structures and cell wall cavities. The structural disintegration weakens the physical retention of water and oil via capillary tension, leading to a decline in WHC and OHC [42]. A similar decrease in WHC and OHC following powder-size reduction has also been reported for *Hericium erinaceus* powders [43], suggesting that disruption of the internal porous structure may be a common consequence of intensive size-reduction processing in mushroom powders.

For the SE group, the diminished retention capacities were primarily driven by the instantaneous high pressure and vaporization shock generated during the steam explosion process [25,44]. Consequently, during the measurement process, increased solubilization of matrix components may promote their transfer into the supernatant during centrifugation, thereby reducing the amount of water retained by the residual fraction and resulting in a lower apparent WHC. Furthermore, structural disruption of the powder matrix may weaken the interactions between the matrix and oil, thereby contributing to the decline in OHC [45].

The WSI of the *M. sextelata* stipe powders differed significantly across the treatments (*p* < 0.05). The WSI increased from 31.20% in CG to 34.79% after UG treatment and further increased to 36.55% in the SE-UG group. Compared with the untreated sample, SE-UG resulted in an approximately 17.15% increase in WSI and showed the largest increase among the four processing groups. The simultaneous decrease in WHC/OHC and increase in WSI reflect different aspects of the modification process. The decline in WHC and OHC may be related to disruption of macromolecular networks and physical retention pores, whereas the elevated WSI is consistent with increased release of soluble components after structural disruption. UG increases the contact area between particles and water and may facilitate the transfer of soluble polysaccharides, low-molecular-weight sugars, and partially hydrolyzed products into the aqueous phase [46]. Previous studies have shown that SE can promote the transformation of structural components and increase the soluble dietary fiber fraction of plant-derived materials [47]. From a practical perspective, the higher WSI may improve dispersion of the stipe powder in aqueous food systems, although the concurrent decreases in WHC and OHC indicate that the treatments did not improve all hydration-related properties.

### 3.6. Fourier-Transform Infrared Spectroscopy (FT-IR) of Morchella sextelata Stipe Powder

**Figure 5:**
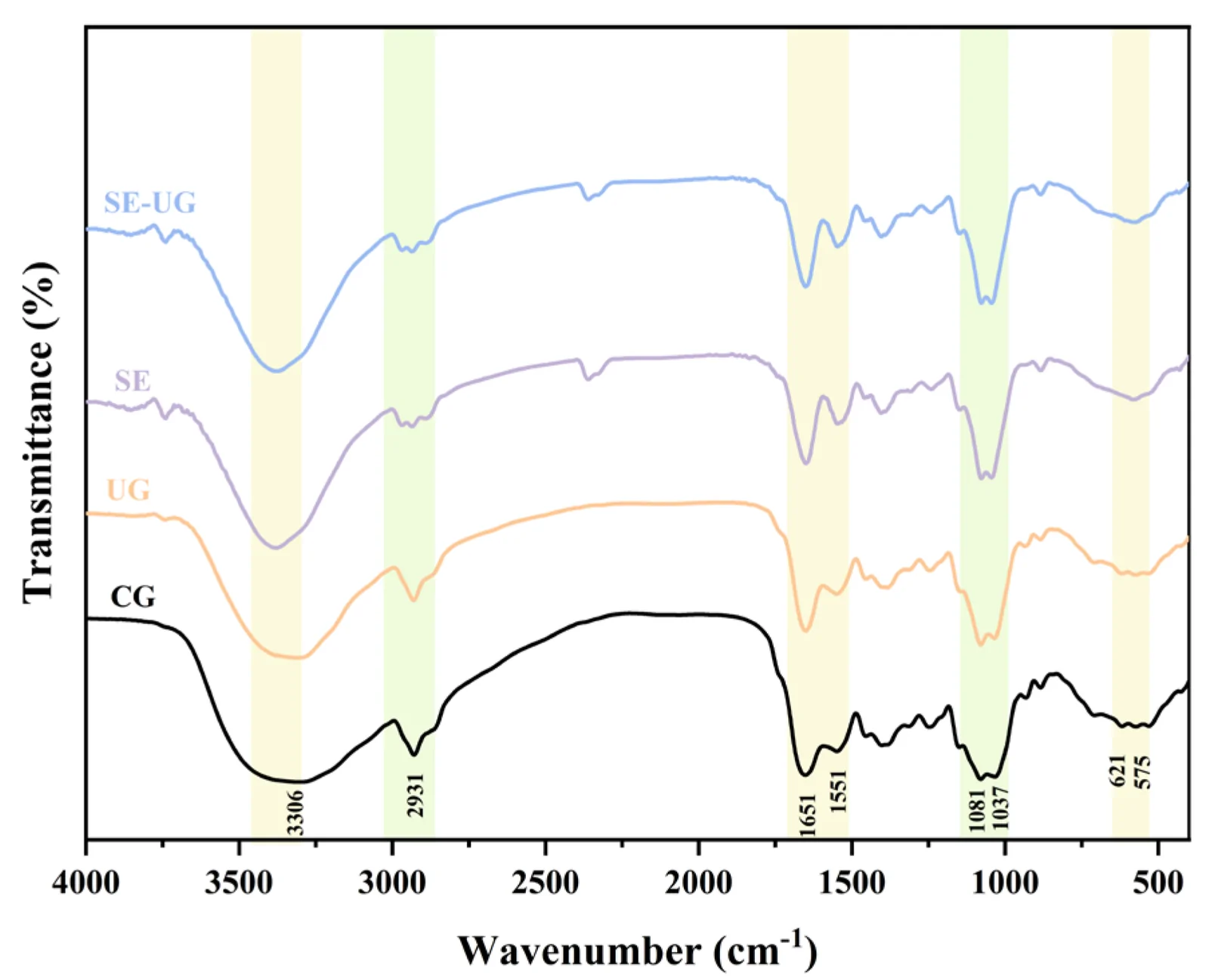
FT-IR spectra of *M. sextelata* stipe powders subjected to different physical modification treatments.

The FT-IR spectra of *M. sextelata* stipe powders with different physical modifications are presented in Figure 5. All samples exhibited similar characteristic absorption bands, suggesting that the modification treatments did not change the primary chemical structures of the stipe powders.

In the FT-IR spectra, the broad absorption band around 3306 cm^−1^ was mainly attributed to the O-H stretching vibration of hydroxyl groups involved in hydrogen bonding [48]. The absorption peak at 2931 cm^−1^ corresponded to the C-H stretching vibration of aliphatic methyl and methylene groups [49]. Two characteristic peaks located at 1651 and 1551 cm^−1^ were assigned to the amide I and amide II bands, respectively. The amide I band was mainly associated with C=O stretching vibrations, whereas the amide II band was related to N-H bending and C-N stretching vibrations of peptide bonds [50]. Moreover, the strong absorption region between 1200 and 1000 cm^−1^ represents the characteristic region of polysaccharides, mainly corresponding to C-O-C and C-O stretching vibrations of glycosidic bonds. The weak absorption bands at 621 and 575 cm^−1^ were related to the skeletal vibrations of pyranose rings in polysaccharide structures [13].

Although the treatments did not alter the major functional groups of *M. sextelata* stipe powders, they might induce changes in structural organization. The high-temperature and high-pressure conditions during steam explosion, together with the mechanical forces generated by ultrafine grinding, could modify hydrogen bonding interactions within the polysaccharide–protein matrix. These alterations may contribute to the loosening of the structural network, thereby improving solvent accessibility and facilitating the release of soluble components during extraction [46].

### 3.7. X-Ray Diffraction Analysis of Morchella sextelata Stipe Powders

The characteristics of diffraction peaks in X-ray diffraction (XRD) patterns provide important information regarding the crystalline structure and the degree of structural order in samples. In this study, XRD analysis was performed to investigate the effects of different physical modifications on the structural characteristics of *M. sextelata* stipe powders.

**Figure 6:**
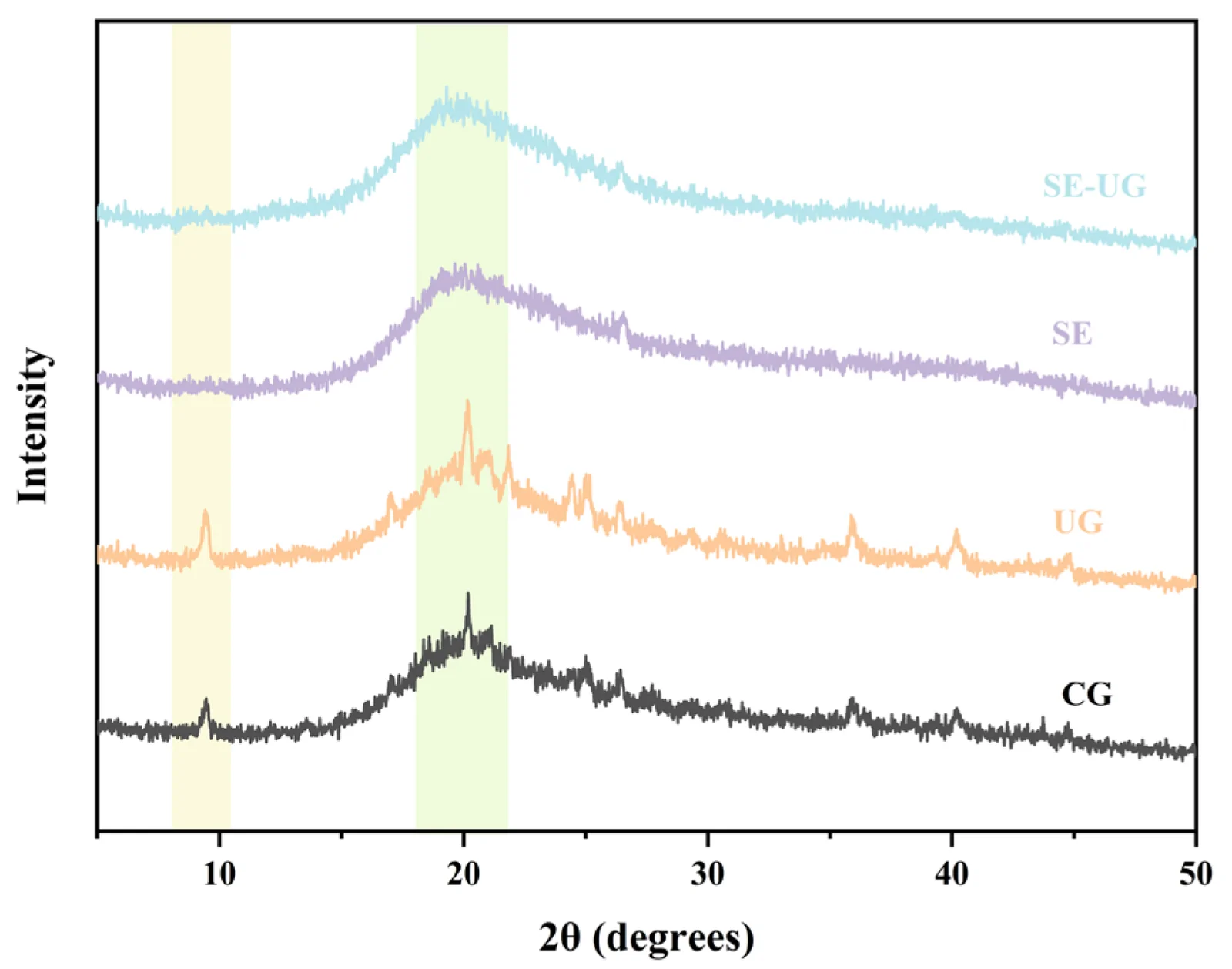
X-ray diffraction patterns of *M. sextelata* stipe powders subjected to different physical modification treatments.

As shown in Figure 6, all samples exhibited a broad diffraction peak around 2θ = 20°, indicating the coexistence of ordered structures and amorphous regions within the stipe powders [13]. This feature may be associated with the heterogeneous composition and partially ordered arrangement of fungal cell wall components. In the CG and UG groups, several weak diffraction peaks were observed at approximately 2θ values of 9°, 20°, 36°, and 40°, with the peak near 20° exhibiting the highest intensity. After steam explosion treatment, the characteristic diffraction signal around 9° was weakened, while the broad peak near 20° became wider with reduced intensity. Consequently, the overall diffraction patterns became flatter compared with those of the CG and UG groups, suggesting that steam explosion treatment disrupted the original ordered structures and promoted the formation of more amorphous regions [51]. The decrease in diffraction intensity indicated the disruption of ordered structures and an increased degree of structural disorder in the stipe powders. These structural changes could be mainly attributed to the high-pressure disruption induced by steam explosion and the subsequent mechanical fragmentation during ultrafine grinding. During the SE process, intense thermal-mechanical stress combined with instantaneous decompression may disrupt the rigid fungal cell wall structure, resulting in the loosening of the original molecular arrangement [52].

### 3.8. Thermogravimetric Analysis

**Figure 7:**
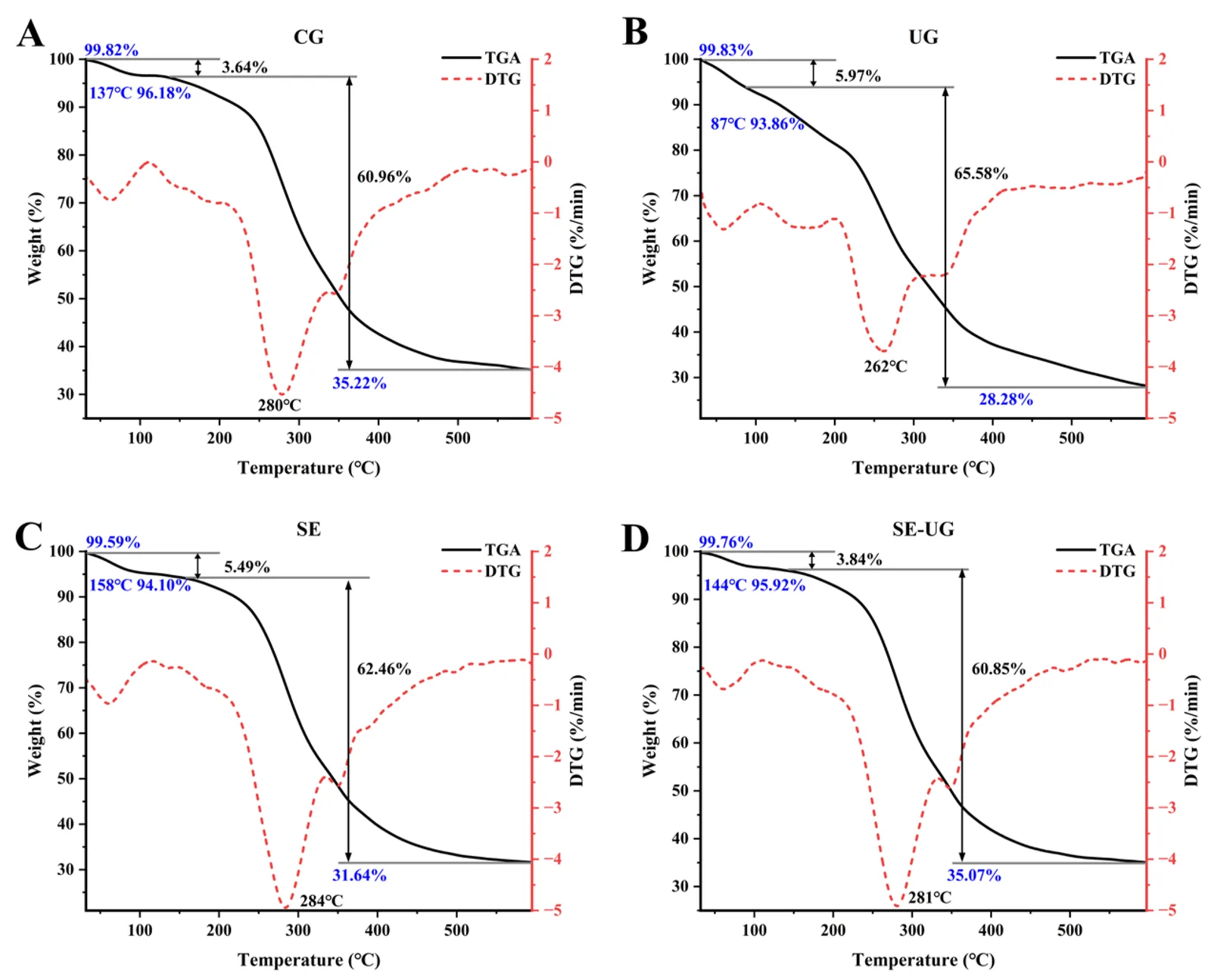
TGA and DTG curves of *M. sextelata* stipe powders subjected to different physical modification treatments. (**A**) CG; (**B**) UG; (**C**) SE; and (**D**) SE-UG.

Thermogravimetric analysis (TGA) and DTG were employed to investigate the thermal degradation behavior and thermal stability of modified *M. sextelata* stipe powders. As shown in Figure 7, all samples exhibited a similar two-stage weight loss pattern. The initial slight weight loss observed below 200 °C was mainly attributed to the evaporation of free water and weakly bound water [53]. The UG group exhibited the highest weight loss (5.97%) during this stage. This phenomenon might be associated with the reduced particle size and increased specific surface area caused by ultrafine grinding, which could enhance moisture adsorption from the surrounding environment [46].

The major degradation stage occurred between 200 °C and 600 °C, which was mainly attributed to the thermal decomposition of organic components, including polysaccharides, proteins, and other macromolecules [54]. The DTG curves showed that ultrafine grinding decreased the maximum degradation temperature (T_max_) from 280 °C (CG) to 262 °C (UG), suggesting reduced thermal stability. This may be associated with the decreased particle size and increased specific surface area after grinding, which could facilitate heat transfer and accelerate thermal degradation. In contrast, steam explosion treatments slightly increased T_max_ values to 284 °C (SE) and 281 °C (SE-UG), possibly due to hydrothermal-induced structural modification and redistribution of heat-sensitive components. Overall, SE and SE-UG treatments exhibited relatively stable thermal behavior before 200 °C, indicating their potential tolerance to moderate thermal processing conditions in food applications [55].

### 3.9. DPPH and ABTS Radical Scavenging Activities

**Figure 8:**
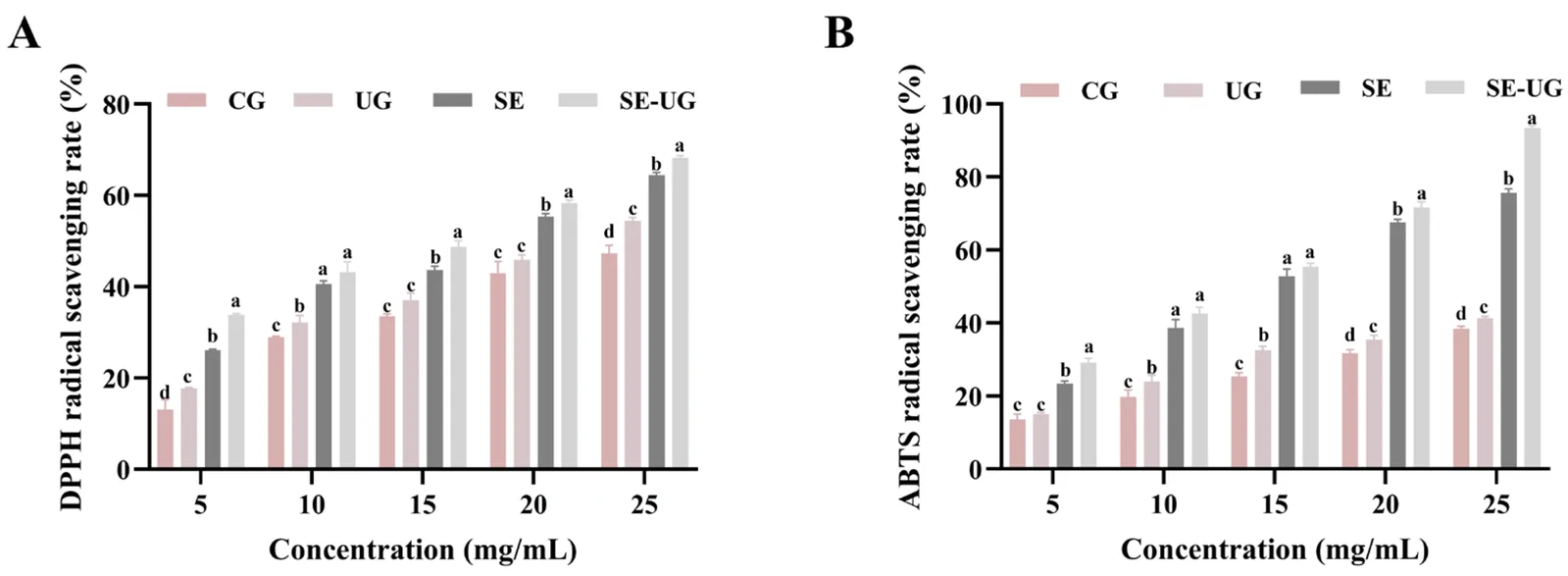
Effects of different physical modification treatments on DPPH (**A**) and ABTS (**B**) radical scavenging activities of *M. sextelata* stipe powders. Different letters at the same concentration indicate significant differences among treatments (*p* < 0.05).

The DPPH and ABTS radical scavenging assays are widely used to evaluate the in vitro antioxidant activity of food materials and their extracts [56]. As shown in Figure 8, the DPPH and ABTS radical scavenging activities of all modified *M. sextelata* stipe powders exhibited a concentration-dependent increase. At the same concentration, different physical modification treatments significantly influenced the radical scavenging activities of the stipe powders (*p* < 0.05).

For the DPPH assay, all modified samples showed higher measured scavenging rates than CG across the tested concentration range. At 25 mg/mL, the DPPH scavenging rates of CG, UG, SE, and SE-UG were approximately 48.66%, 54.59%, 64.42%, and 68.70%, respectively. A similar concentration-dependent pattern was observed for ABTS; at 30 mg/mL, the ABTS scavenging rates were 38.61%, 41.59%, 75.87%, and 93.35% for CG, UG, SE, and SE-UG, respectively. The SE-UG group therefore showed the highest measured DPPH and ABTS responses under the tested conditions. Because a 50% scavenging response was not reached within the tested concentration range for all groups (CG for DPPH; CG and UG for ABTS), IC_50_ values were not calculated in order to avoid extrapolation beyond the measured range. Comparisons are therefore based on the observed concentration-response profiles and measured scavenging rates.

The higher antioxidant responses observed in SE and SE-UG-treated samples may be associated with disruption of the compact cell-wall structure and improved extractability of antioxidant-active constituents. Steam explosion has been reported to promote the liberation of bound phenolics and other bioactive compounds in plant matrices. For example, steam explosion pretreatment enhanced phenolic composition and antioxidant capacity in tartary buckwheat bran and mung beans, effects attributed to improved accessibility of antioxidant compounds after structural disruption [57,58]. Similar enhancement of antioxidant properties following steam explosion treatment has also been observed in raspberry leaves, further supporting the role of thermal-mechanical pretreatment in improving bioactive compound extraction [59]. In the present study, the subsequent ultrafine grinding treatment further increased the specific surface area of stipe powders [60], which may facilitate the release and extraction of antioxidant components, resulting in the superior scavenging capacity of the SE-UG group.

### 3.10. Total Antioxidant Capacity of Modified Morchella sextelata Stipe Powders

**Figure 9:**
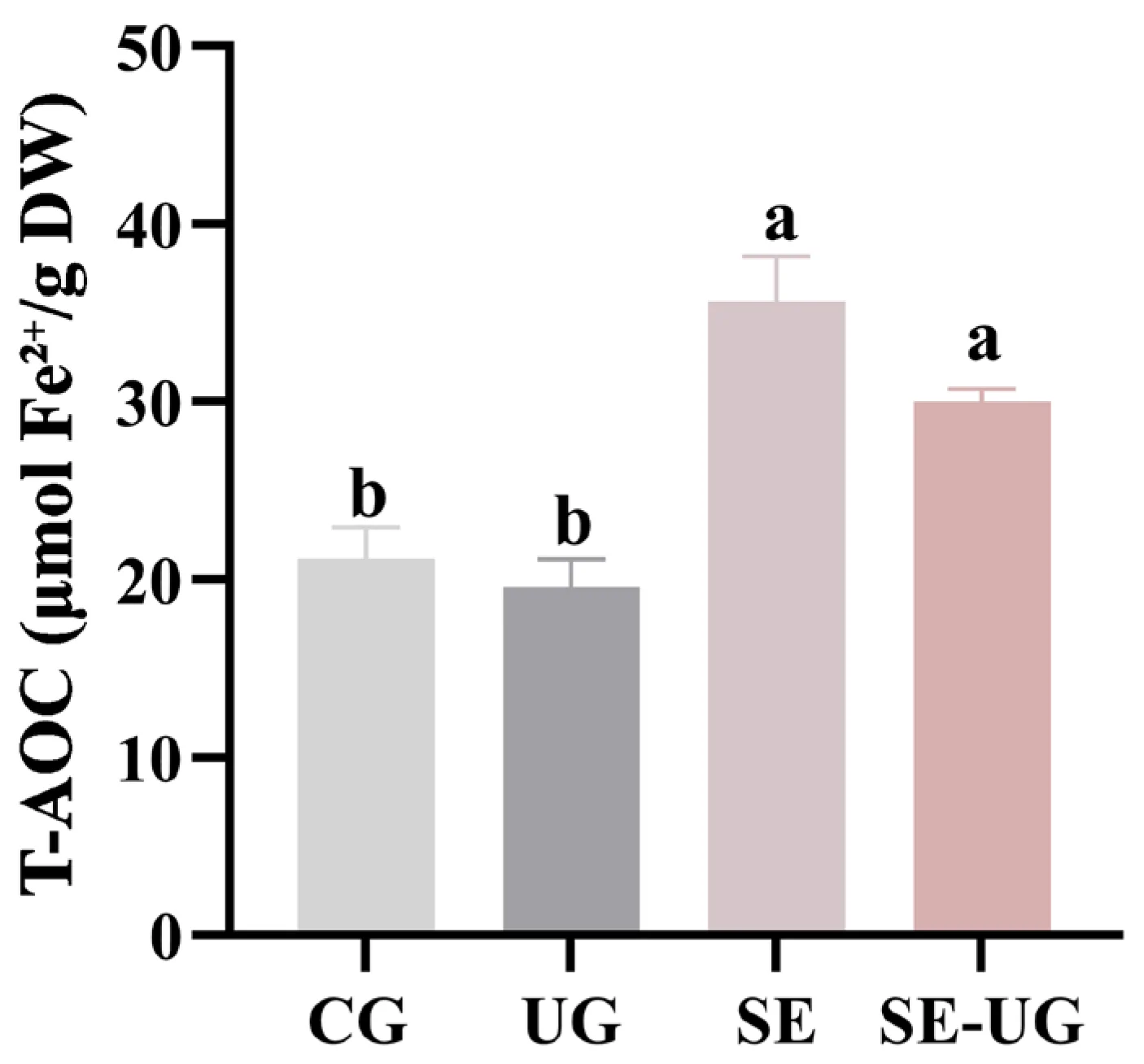
T-AOC of *M. sextelata* stipe powders. Different lowercase letters indicate significant differences among treatments (*p* < 0.05).

T-AOC is commonly used to evaluate the overall reducing ability of antioxidant constituents in food matrices [61]. As shown in Figure 9, different physical modification treatments significantly affected the T-AOC of *M. sextelata* stipe powders (*p* < 0.05). The CG and UG groups exhibited relatively lower T-AOC values and did not differ significantly from each other. In contrast, both SE and SE-UG exhibited significantly higher T-AOC than CG and UG. Although their numerical values were 35.6 and 30.1 μmol Fe^2+^/g DW, respectively, SE and SE-UG shared the same significance group and therefore did not differ significantly from each other. Accordingly, the numerical difference should not be interpreted as evidence that SE had a statistically greater T-AOC than SE-UG. Comparable differences in antioxidant capacity have also been observed among edible mushroom species and tissues. Wang et al. investigated the effects of stem and cap fractions from white button mushroom (*Agaricus bisporus*) and oyster mushroom (*Pleurotus ostreatus*) incorporated into wheat noodles. Their results showed differences in antioxidant capacity among the mushroom-fortified noodle systems and a positive correlation between mushroom-derived insoluble dietary fiber and antioxidant measures, suggesting that mushroom tissue composition may influence the antioxidant properties of food systems [62].

Similar enhancement of antioxidant properties following steam explosion-assisted superfine grinding has been reported previously, which was associated with changes in the physical structure and chemical composition of the treated material [16]. The higher reducing capacity in the SE-containing groups may be associated with structural disruption and improved accessibility of reducing compounds. Previous studies have shown that steam explosion can enhance total antioxidant capacity, which may be related, at least in part, to the increased release of soluble phenolic compounds [63]. The discrepancy between radical-scavenging assays and T-AOC may reflect differences in assay mechanisms and the compounds detected by each analytical system [61].

The enhanced T-AOC values observed in the SE and SE-UG groups may be associated with the disruption of the compact fungal cell wall structure and the subsequent release of intracellular reducing compounds. Similar effects have been reported in plant-derived materials, where steam explosion promoted the liberation of phenolic compounds and improved antioxidant capacity by enhancing cell wall permeability and inducing structural degradation [64,65]. In addition, thermal reactions during steam explosion may also contribute to changes in the reducing capacity of the modified powders.

### 3.11. Effect of Physical Modifications on the Volatile Profiles of Morchella sextelata Stipe Powders Based on HS-SPME-GC-MS Analysis

**Figure 10:**
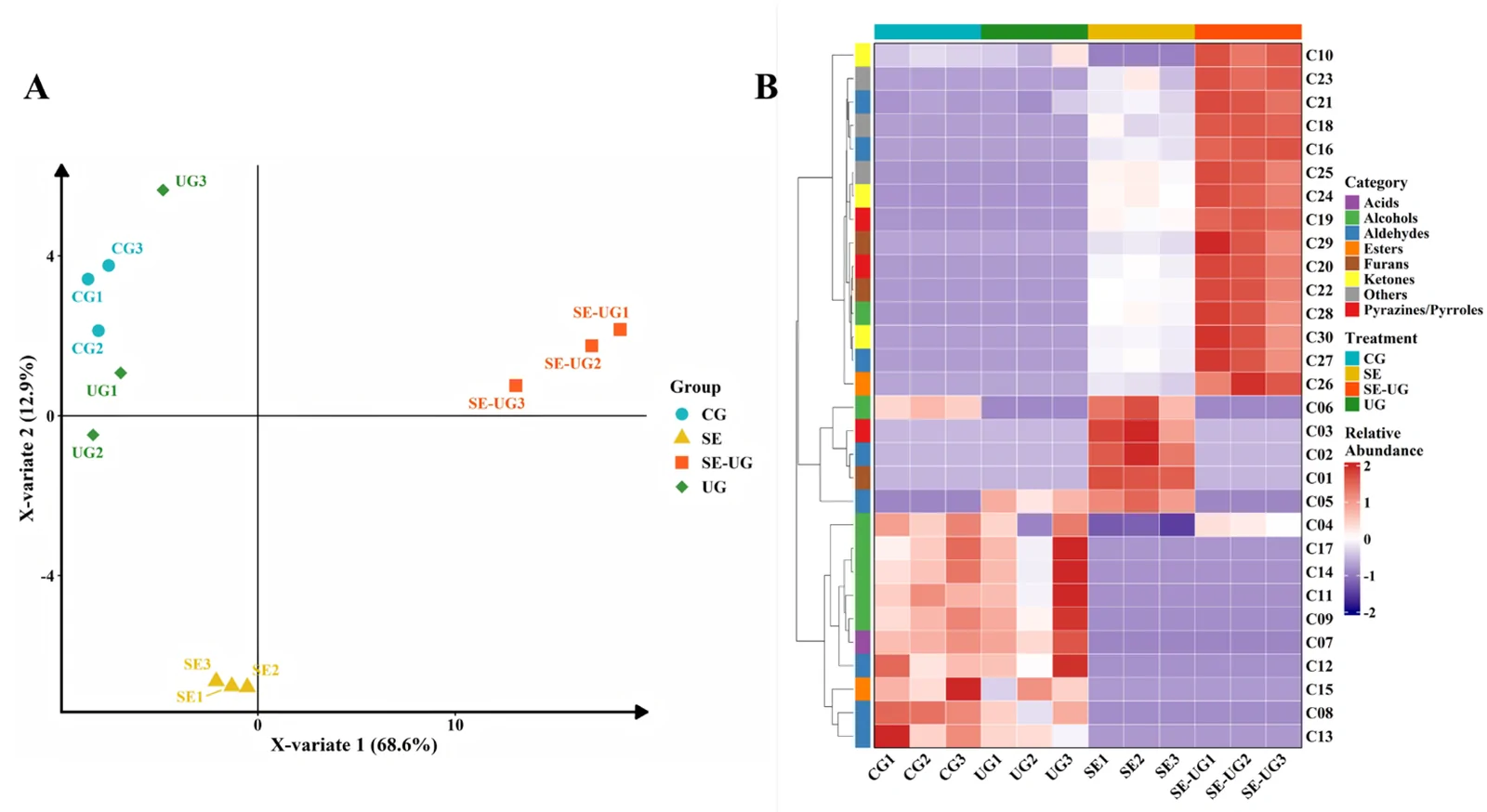
(**A**) PLS-DA score plot of volatile compounds in *M. sextelata* stipe powders subjected to different physical modification treatments. (**B**) Heatmap and hierarchical clustering analysis of the 30 differential volatile compounds.

To characterize differences in volatile profiles among differently processed *M. sextelata* stipe powders, partial least squares discriminant analysis (PLS-DA) was performed based on the relative abundances of the detected volatile compounds. As shown in Figure 10A, the four treatment groups exhibited distinct distribution patterns in the PLS-DA score plot. The first two latent variables (LV1 and LV2) explained 68.6% and 12.9% of the total variance, respectively. Replicates within each treatment group were closely clustered, indicating good within-group consistency [66]. Seven-fold cross-validation with 10 repeats was further performed to evaluate the classification performance of the PLS-DA model. Using two components and the maximum-distance classification criterion, the cross-validated overall error rate was approximately 27.5%. In addition, a 200-permutation test was conducted by randomly permuting the class labels. The observed balanced error rate (BER) was 0.4417, whereas only 2 of the 200 permuted models produced a BER equal to or lower than the observed value, corresponding to a permutation P value of 0.0149. These results support a non-random association between processing treatment and the observed differences in volatile profiles [67]. The CG and UG groups occupied relatively similar regions in the score plot, indicating that ultrafine grinding alone was associated with comparatively limited changes in the overall volatile profile [46]. In comparison, the SE and SE-UG groups were clearly separated from CG and UG, suggesting that treatments involving steam explosion were associated with more pronounced changes in the overall distribution of volatile compounds. The further separation between SE and SE-UG also indicates that ultrafine grinding following steam explosion was associated with an additional shift in volatile composition.

HCA was subsequently conducted using 30 key differential volatile compounds selected according to their variable importance in projection (VIP) scores (Table 3) to further examine the distribution patterns of volatile compounds among treatments. As shown in Figure 10B, the CG and UG samples clustered together, whereas the SE and SE-UG samples formed a separate cluster, in agreement with the PLS-DA results. This clustering pattern further demonstrates that the volatile profiles of the SE-containing treatments differed more substantially from those of CG and UG, while ultrafine grinding alone produced comparatively smaller changes in the overall volatile composition [18]. The heatmap further showed treatment-dependent differences in the relative abundance patterns of individual volatile compounds. The complete list of volatile compounds and their relative abundances under different treatments is provided in Appendix A (Table A1). Alcohols and several aldehydes were predominantly associated with the CG and UG samples. Among these compounds, 1-hexanol, 3-methyl-1-butanol, and 1-octanol have previously been associated with green, herbaceous, and mushroom-like odor characteristics in edible fungi [68]. Their lower relative abundances in the SE and SE-UG groups may be related to differences in their stability, volatility, or transformation behavior under the thermal conditions associated with steam explosion [69]. Notably, 1-octen-3-ol, a characteristic volatile compound widely associated with mushroom aroma, was detected in all four treatment groups and exhibited a markedly higher relative abundance in the SE-UG group than in the other groups. This distribution indicates that the sequential SE-UG treatment was associated not only with changes in thermally related volatile compounds but also with the relative abundance of characteristic mushroom-associated volatiles. In contrast, several furan derivatives and pyrazines exhibited higher relative abundances in the SE and SE-UG groups. Such changes are consistent with volatile formation pathways involving carbohydrate degradation and Maillard- or Strecker-related reactions under thermal processing conditions [70]. The elevated temperature and pressure applied during steam explosion may have favored reactions involving reducing sugars and amino acid-derived precursors, which could contribute to the increased relative abundance of furan and pyrazine derivatives. These classes of compounds are commonly associated with roasted, nutty, or cooked aroma characteristics [71].

Several ketones also showed higher relative abundances in the SE-UG group. These changes may be related to alterations in lipid-derived volatile pathways and to differences in the release or accessibility of volatile compounds and their precursors following the combined SE and UG treatments [72,73]. Overall, the multivariate and compound-level analyses consistently indicate that steam explosion was associated with a greater reorganization of the volatile profile than ultrafine grinding alone, while subsequent ultrafine grinding following steam explosion further modified the relative distribution of selected volatile compounds.

**Table 3:** Key tentatively identified volatile compounds ranked by VIP scores among differently processed *M. sextelata* stipe powders.

Compound ID	Compound	Aroma Description	VIP Value
C01	3-Furaldehyde	Sweet	2.051
C02	Pentanal, 2,4-dimethyl-	Green	2.033
C03	Ethanone, 1-(3,5-dimethylpyrazinyl)-	Roasted	1.986
C04	1-Hexanol	Green	1.728
C05	Butanal, 3-methyl-	Malty	1.602
C06	2H-Pyranmethanol, tetrahydro-2,5-dimethyl-	Sweet	1.454
C07	Pentanoic acid, 3-methyl-	Cheese	1.403
C08	Nonanal	Fatty	1.394
C09	1-Butanol, 3-methyl-	Malty	1.319
C10	5,9-Undecadien-2-one, 6,10-dimethyl-, (E)-	Floral	1.292
C11	1-Octanol	Fatty	1.288
C12	2-Nonenal, (E)-	Green	1.281
C13	Dodecanal	Citrus	1.268
C14	1-Propanol, 2-methyl-	Fruity	1.255
C15	Acetic acid, hexyl ester	Fruity	1.234
C16	2-Ethylacrolein	Green	1.218
C17	1-Heptanol	Green	1.217
C18	1,5-Dimethyl-2-pyrrolecarbonitrile	Roasted	1.214
C19	Pyrazine, 2-methyl-3-(2-propenyl)-	Roasted	1.211
C20	1H-Pyrrole-2-carboxaldehyde	Roasted	1.21
C21	2-Butenal	Green	1.208
C22	2,5-Dimethylfuran-3,4(2H,5H)-dione	Sweet	1.208
C23	Ethanone, 1-(3-thienyl)-	Roasted	1.204
C24	1,4-Cyclohex-2-enedione	Sweet	1.203
C25	2-Acetylthiazole	Roasted	1.201
C26	Ethyl Acetate	Fruity	1.201
C27	Benzeneacetaldehyde, α-ethylidene-	Floral	1.2
C28	2-Propyl-1-pentanol	Fatty	1.198
C29	trans-Furfurylideneacetone	Caramel	1.198
C30	2-Cyclopenten-1-one, 3-ethyl-2-hydroxy-	Roasted	1.197

### 3.12. Challenges, Limitations and Future Perspectives

Despite the advantages of the SE-UG treatment observed in this study, several challenges and limitations should be considered. First, the effectiveness of steam explosion is highly dependent on processing parameters, and the conditions used in the present study were not further optimized specifically for *M. sextelata* stipes. Future studies should therefore systematically optimize key steam explosion parameters, such as pressure, residence time, and moisture content, to determine the optimal conditions for different types of morel stipes. Second, the SE-UG process involves additional moisture conditioning and drying steps, which may increase processing complexity and energy requirements; thus, pilot-scale validation and techno-economic evaluation are needed to assess its feasibility for scale-up [74]. In addition, comparative studies using different *Morchella* species and stipe materials from different varieties or production sources would be valuable for determining whether differences in species, cultivar, or raw-material characteristics affect the response to SE-UG treatment. Furthermore, the mechanisms underlying the observed changes in antioxidant capacity and physicochemical properties remain to be clarified at the level of specific bioactive components. Future studies should investigate whether SE-UG promotes the release, degradation, or structural transformation of bioactive compounds, particularly polysaccharides and phenolic compounds, and further elucidate their contributions to the observed antioxidant effects. Finally, targeted volatile analysis combined with sensory evaluation and odor activity value analysis could provide a more comprehensive understanding of the sensory implications of the volatile changes induced by SE-UG.

## 4. Conclusions

In this study, SE, UG, and their sequential combination (SE-UG) induced distinct changes in the physicochemical characteristics, antioxidant capacity, and relative headspace volatile profiles of *M. sextelata* stipe powders. The SE-containing treatment routes were associated with disruption of the compact stipe matrix, while subsequent UG further reduced particle size and increased specific surface area. Among the treatments, SE-UG resulted in the smallest median particle size and the highest WSI, whereas WHC and OHC decreased, indicating that the combined treatment did not improve all hydration-related properties. SE-UG exhibited the highest measured DPPH and ABTS radical-scavenging activities, while SE and SE-UG showed similarly high T-AOC values, with no significant difference between the two treatments. HS-SPME-GC-MS combined with exploratory multivariate analysis revealed clear treatment-associated differences in relative headspace volatile profiles, with several tentatively identified furan derivatives and pyrazines showing higher relative abundances in the SE-containing groups. Overall, sequential SE followed by UG effectively modified selected physicochemical and antioxidant properties and altered the relative volatile profiles of *M. sextelata* stipe powders. These findings provide a basis for further process optimization and support the potential value-added utilization of mushroom stipe by-products.

## Figures and Tables

**Table 1:** Particle size distribution parameters of *M. sextelata* stipe powders subjected to different physical modification treatments.

	CG	UG	SE	SE-UG
D_10_ ( μm )	16.92 ± 0.31 ^a^	12.35 ± 0.33 ^b^	7.03 ± 0.36 ^c^	3.53 ± 0.03 ^d^
D_50_ ( μm )	88.88 ± 1.10 ^a^	44.39 ± 1.40 ^b^	33.87 ± 3.26 ^c^	10.46 ± 0.26 ^d^
D_90_ ( μm )	172.65 ± 0.40 ^a^	101.15 ± 1.32 ^b^	148.94 ± 10.40 ^a^	24.47 ± 0.17 ^c^
D_[3,2]_ ( μm )	35.24 ± 0.37 ^a^	24.91 ± 0.67 ^b^	16.44 ± 0.91 ^c^	7.27 ± 0.06 ^d^
D_[4,3]_ ( μm )	92.16 ± 0.66 ^a^	51.09 ± 1.13 ^b^	56.78 ± 5.06 ^b^	12.51 ± 0.09 ^c^
S/V (cm^2^/cm^3^)	1702.71 ± 17.93 ^d^	2409.96 ± 65.36 ^c^	3656.65 ± 206.51 ^b^	8256.48 ± 70.17 ^a^
Span	1.752 ± 0.020 ^c^	2.002 ± 0.042 ^b^	4.198 ± 0.41 ^a^	1.968 ± 0.012 ^b^

Different lowercase superscript letters within the same row indicate significant differences among treatments (*p* < 0.05).

**Table 2:** Effect of different modification methods on the color of *M. sextelata* stipe.

Samples	L*	a*	b*	Δ*E*
CG	80.41 ± 0.29 ^a^	4.63 ± 0.11 ^c^	17.60 ± 0.20 ^b^	0.00 ± 0.00 ^d^
UG	79.50 ± 0.16 ^b^	2.8 ± 0.02 ^d^	16.08 ± 0.08 ^c^	2.63 ± 0.06 ^c^
SE	68.76 ± 0.25 ^d^	6.72 ± 0.03 ^a^	19.74 ± 0.17 ^a^	11.70 ± 0.21 ^a^
SE-UG	75.13 ± 0.12 ^c^	5.24 ± 0.08 ^b^	17.95 ± 0.19 ^b^	5.03 ± 0.13 ^b^

Different lowercase superscript letters within the same column indicate significant differences among treatments (*p* < 0.05).

## Data Availability

The original contributions presented in this study are included in the article. Further inquiries can be directed to the corresponding authors.

## Abbreviations

*M. sextelata*	*Morchella sextelata*
CG	Conventional Grinding
UG	Ultrafine Grinding
SE	Steam Explosion
SE-UG	Steam Explosion–Ultrafine Grinding
SEM	Scanning Electron Microscopy
FT-IR	Fourier-Transform Infrared Spectroscopy
XRD	X-ray Diffraction
WHC	Water-Holding Capacity
OHC	Oil-Holding Capacity
WSI	Water Solubility Index
TG	Thermogravimetric
DTG	Differential Thermogravimetric
TGA	Thermogravimetric Analysis
DSC	Differential Scanning Calorimetry
TGA-DSC	Thermogravimetric Analysis–Differential Scanning Calorimetry
DPPH	2,2-Diphenyl-1-picrylhydrazyl
ABTS	2,2′-Azino-bis(3-ethylbenzothiazoline-6-sulfonic acid)
T-AOC	Total Antioxidant Capacity
DW	Dry Weight
HS-SPME	Headspace Solid-Phase Microextraction
GC	Gas Chromatography
MS	Mass Spectrometry
GC-MS	Gas Chromatography–Mass Spectrometry
DVB	Divinylbenzene
CAR	Carboxen
PDMS	Polydimethylsiloxane
NIST	National Institute of Standards and Technology
PLS-DA	Partial Least Squares Discriminant Analysis
VIP	Variable Importance in Projection
HCA	Hierarchical Cluster Analysis
LV1	Latent Variable 1
LV2	Latent Variable 2
BER	Balanced Error Rate
ND	Not Detected

## Appendix A

**Table A1:** Volatile compounds and their relative abundance in *M. sextelata* stipe powders under different treatments.

NO.	Name	CAS	Chemical Class	CG	UG	SE	SE-UG
1	1-Propanol, 2-methyl-	78-83-1	Alcohol	0.27 ± 0.10	0.3 ± 0.21	ND	ND
2	1-Butanol, 3-methyl-	123-51-3	Alcohol	0.95 ± 0.35	1.18 ± 0.81	ND	ND
3	1-Pentanol	71-41-0	Alcohol	0.75 ± 0.21	0.77 ± 0.56	0.44 ± 0.09	1.44 ± 0.13
4	1-Hexanol, 5-methyl-2-(1-methylethyl)-	2051-33-4	Alcohol	0.01 ± 0.00	ND	0.02 ± 0.00	0.06 ± 0.01
5	1-Hexanol	111-27-3	Alcohol	3.24 ± 0.69	2.48 ± 0.58	0.56 ± 0.10	2.04 ± 0.22
6	2H-Pyranmethanol, tetrahydro-2,5-dimethyl-	54004-46-5	Alcohol	0.02 ± 0.00	ND	0.03 ± 0.01	ND
7	1-Octen-3-ol	3391-86-4	Alcohol	1.39 ± 0.40	1.72 ± 0.21	1.74 ± 0.60	8.96 ± 1.15
8	1-Heptanol	111-70-6	Alcohol	0.09 ± 0.04	0.11 ± 0.07	ND	ND
9	Linalool	78-70-6	Alcohol	1.59 ± 0.09	ND	ND	ND
10	-5-Methyl-2-(prop-1-en-2-yl)cyclohexanol	29141-10-4	Alcohol	1.49 ± 0.02	ND	ND	ND
11	1-Octanol	111-87-5	Alcohol	0.24 ± 0.05	0.26 ± 0.10	ND	ND
12	2,3-Butanediol	513-85-9	Alcohol	0.05 ± 0.01	0.06 ± 0.01	ND	0.37 ± 0.05
13	2-Octen-1-ol	18409-17-1	Alcohol	0.11 ± 0.01	0.13 ± 0.02	0.09 ± 0.02	0.62 ± 0.09
14	4-Nonanol	5932-79-6	Alcohol	0.09 ± 0.00	0.35 ± 0.06	ND	ND
15	1-Nonen-4-ol	35192-73-5	Alcohol	0.17 ± 0.02	ND	ND	ND
16	Benzyl alcohol	100-51-6	Alcohol	0.84 ± 0.02	0.44 ± 0.03	0.53 ± 0.08	1.70 ± 0.01
17	Phenylethyl Alcohol	60-12-8	Alcohol	0.15 ± 0.03	0.21 ± 0.04	0.16 ± 0.03	0.68 ± 0.05
18	1-Dodecanol	112-53-8	Alcohol	ND	0.06 ± 0.01	0.14 ± 0.03	0.47 ± 0.07
19	2-Propyl-1-pentanol	58175-57-8	Alcohol	ND	ND	0.45 ± 0.06	2.07 ± 0.05
20	2,6-Heptadien-1-ol, 2,4-dimethyl-	80192-56-9	Alcohol	ND	ND	0.06 ± 0.01	ND
21	2,3-Butanediol	24347-58-8	Alcohol	ND	0.13 ± 0.04	ND	ND
22	1,6,10-Dodecatrien-3-ol, 3,7,11-trimethyl-	13925-03-6	Alcohol	ND	ND	ND	0.15 ± 0.03
23	Pentanal	110-62-3	Aldehyde	0.44 ± 0.02	0.02 ± 0.00	0.31 ± 0.06	1.41 ± 0.04
24	2-Butenal	4170-30-3	Aldehyde	0.05 ± 0.01	0.08 ± 0.06	0.20 ± 0.03	0.80 ± 0.07
25	Hexanal	66-25-1	Aldehyde	2.93 ± 0.03	3.24 ± 0.07	2.13 ± 0.01	9.52 ± 0.01
26	2-Hexenal	6728-26-3	Aldehyde	1.06 ± 0.09	0.11 ± 0.06	ND	ND
27	2-Pentenal, 2-methyl-	623-36-9	Aldehyde	0.02 ± 0.01	0.02 ± 0.01	ND	ND
28	Octanal	124-13-0	Aldehyde	0.23 ± 0.01	0.16 ± 0.01	0.18 ± 0.05	0.75 ± 0.03
29	Nonanal	124-19-6	Aldehyde	2.25 ± 0.07	0.96 ± 0.04	ND	ND
30	Citronellal	106-23-0	Aldehyde	3.70 ± 0.11	ND	ND	ND
31	6-Octenal, 3,7-dimethyl-	2385-77-5	Aldehyde	0.45 ± 0.08	ND	ND	ND
32	Benzaldehyde	100-52-7	Aldehyde	7.24 ± 0.05	2.05 ± 0.06	3.41 ± 0.18	15.55 ± 0.19
33	2-Nonenal	18829-56-6	Aldehyde	0.35 ± 0.05	0.37 ± 0.05	ND	ND
34	2-Butylhept-2-enal	73757-26-3	Aldehyde	0.12 ± 0.03	0.17 ± 0.06	ND	ND
35	2-Octenal, 2-butyl-	13019-16-4	Aldehyde	0.55 ± 0.10	1.20 ± 0.03	ND	ND
36	Dodecanal	112-54-9	Aldehyde	0.13 ± 0.06	0.06 ± 0.02	ND	ND
37	Butanal, 3-methyl-	590-86-3	Aldehyde	ND	1.66 ± 0.09	2.92 ± 0.06	ND
38	Pentanal, 2,4-dimethyl-	27944-79-2	Aldehyde	ND	0.13 ± 0.01	0.11 ± 0.02	ND
39	2-Ethylacrolein	922-63-4	Aldehyde	ND	ND	0.10 ± 0.01	0.49 ± 0.02
40	2,4-Decadienal	25152-84-5	Aldehyde	ND	ND	0.07 ± 0.02	0.35 ± 0.07
41	Benzeneacetaldehyde, .alpha.-ethylidene-	4411-89-6	Aldehyde	ND	ND	0.21 ± 0.03	0.90 ± 0.02
42	Butanal	123-72-8	Aldehyde	ND	0.02 ± 0.00	ND	0.01 ± 0.00
43	2-Hexenal, 2-ethyl-	645-62-5	Aldehyde	ND	0.22 ± 0.04	ND	ND
44	Heptanal	111-71-7	Aldehyde	ND	ND	ND	0.81 ± 0.09
45	2-n-Butylacrolein	1070-66-2	Aldehyde	ND	0.05 ± 0.04	ND	0.11 ± 0.01
46	2-Hexenal	505-57-7	Aldehyde	ND	ND	ND	0.28 ± 0.03
47	Tridecanal	10486-19-8	Aldehyde	ND	ND	ND	0.60 ± 0.09
48	Acetic acid	64-19-7	Acids	1.31 ± 0.04	1.94 ± 0.09	2.51 ± 0.01	11.42 ± 0.18
49	Propanoic acid	1192-58-1	Acids	0.07 ± 0.01	0.09 ± 0.05	0.12 ± 0.04	0.66 ± 0.08
50	Propanoic acid, 2-methyl-	156564-41-9	Acids	0.94 ± 0.03	0.95 ± 0.09	0.77 ± 0.09	2.81 ± 0.05
51	Butanoic acid, 4-hydroxy-	591-81-1	Acids	0.83 ± 0.04	1.40 ± 0.03	0.88 ± 0.11	3.47 ± 0.12
52	Butanoic acid	107-92-6	Acids	0.71 ± 0.06	1.30 ± 0.06	0.88 ± 0.13	3.48 ± 0.13
53	Pentanoic acid, 3-methyl-	105-43-1	Acids	4.36 ± 0.12	5.77 ± 0.10	ND	ND
54	Pentanoic acid	109-52-4	Acids	0.61 ± 0.02	0.81 ± 0.02	0.68 ± 0.09	2.94 ± 0.12
55	Hexanoic acid, 2-methyl-	4536-23-6	Acids	0.18 ± 0.04	0.24 ± 0.09	0.18 ± 0.09	1.80 ± 0.11
56	Pentanoic acid, 4-methyl-	646-7-1	Acids	0.10 ± 0.02	0.13 ± 0.12	0.17 ± 0.03	0.73 ± 0.13
57	Hexanoic acid	142-62-1	Acids	8.77 ± 0.08	12.08 ± 0.02	9.60 ± 0.08	36.81 ± 0.13
58	Heptanoic acid	111-14-8	Acids	0.76 ± 0.08	0.91 ± 0.03	0.91 ± 0.09	3.79 ± 0.11
59	Octanoic acid	124-7-2	Acids	0.56 ± 0.13	0.73 ± 0.09	0.71 ± 0.06	3.51 ± 0.08
60	Nonanoic acid	112-5-0	Acids	1.50 ± 0.03	0.88 ± 0.05	0.86 ± 0.03	3.06 ± 0.01
61	Butanoic acid, 3-methyl-	503-74-2	Acids	ND	ND	4.90 ± 0.07	16.95 ± 0.09
62	2-Octenoic acid	1470-50-4	Acids	ND	0.07 ± 0.05	0.06 ± 0.02	0.34 ± 0.07
63	1-Butanol, 3-methyl-, acetate	123-92-2	Esters	0.01 ± 0.01	ND	ND	ND
64	Hexanoic acid, ethyl ester	123-66-0	Esters	0.25 ± 0.08	0.05 ± 0.04	0.02 ± 0.02	0.01 ± 0.00
65	Acetic acid, hexyl ester	142-92-7	Esters	0.04 ± 0.02	0.03 ± 0.02	ND	ND
66	n-Caproic acid vinyl ester	3050-69-9	Esters	0.11 ± 0.02	ND	0.16 ± 0.03	0.76 ± 0.01
67	2(3H)-Furanone, 5-ethyldihydro-	695-6-7	Esters	0.23 ± 0.03	0.27 ± 0.11	0.17 ± 0.02	ND
68	2(5H)-Furanone	497-23-4	Esters	0.31 ± 0.05	0.31 ± 0.05	2.49 ± 0.07	10.58 ± 0.16
69	2H-Pyran-2-one, tetrahydro-	542-28-9	Esters	0.08 ± 0.02	0.17 ± 0.10	0.30 ± 0.03	1.67 ± 0.05
70	2H-Pyran-2-one, 5,6-dihydro-	3393-45-1	Esters	0.40 ± 0.08	0.68 ± 0.05	0.88 ± 0.12	0.45 ± 0.16
71	2(3H)-Furanone, 5-butyldihydro-	104-50-7	Esters	0.03 ± 0.02	0.07 ± 0.04	0.07 ± 0.01	0.31 ± 0.08
72	2(3H)-Furanone, dihydro-5-pentyl-	104-61-0	Esters	0.24 ± 0.03	0.35 ± 0.05	0.25 ± 0.04	1.23 ± 0.03
73	Ethyl Acetate	141-78-6	Esters	ND	ND	0.01 ± 0.00	0.05 ± 0.01
74	Hexanoic acid, 2-propenyl ester	123-68-2	Esters	ND	0.07 ± 0.01	ND	ND
75	Methional	3268-49-3	Sulfur compounds	0.02 ± 0.00	ND	ND	ND
76	Benzothiazole	95-16-9	Sulfur compounds	0.10 ± 0.08	ND	0.01 ± 0.00	ND
77	2-Acetylthiazole	24295-3-2	Sulfur compounds	ND	ND	0.53 ± 0.08	2.11 ± 0.09
78	Thiazole	288-47-1	Sulfur compounds	ND	ND	0.01 ± 0.00	0.04 ± 0.01
79	Ethanone, 1-(3-thienyl)-	1468-83-3	Sulfur compounds	ND	ND	0.40 ± 0.08	2.71 ± 0.06
80	Pyrazine, methyl-	109-8-0	Pyrazines	0.48 ± 0.02	0.42 ± 0.03	4.84 ± 0.05	14.44 ± 0.04
81	Pyrazine, 2,5-dimethyl-	123-32-0	Pyrazines	1.32 ± 0.06	1.79 ± 0.11	8.42 ± 0.07	24.90 ± 0.03
82	Pyrazine, 2,6-dimethyl-	108-50-9	Pyrazines	0.78 ± 0.09	0.86 ± 0.05	6.65 ± 0.08	17.20 ± 0.08
83	Pyrazine, ethyl-	13925-0-3	Pyrazines	0.14 ± 0.05	0.11 ± 0.06	1.29 ± 0.04	3.66 ± 0.03
84	Pyrazine, 2,3-dimethyl-	5910-89-4	Pyrazines	0.31 ± 0.05	0.36 ± 0.03	0.86 ± 0.04	2.76 ± 0.08
85	Pyrazine, 2-ethyl-6-methyl-	13925-3-6	Pyrazines	0.24 ± 0.09	0.29 ± 0.08	2.25 ± 0.07	5.79 ± 0.06
86	Pyrazine, trimethyl-	14667-55-1	Pyrazines	2.12 ± 0.03	1.91 ± 0.05	4.83 ± 0.09	14.45 ± 0.18
87	Pyrazine, 3-ethyl-2,5-dimethyl-	13360-65-1	Pyrazines	0.35 ± 0.09	0.28 ± 0.10	2.85 ± 0.02	7.64 ± 0.07
88	Pyrazine, 2,6-diethyl-	13067-27-1	Pyrazines	0.25 ± 0.02	0.40 ± 0.07	1.13 ± 0.08	3.43 ± 0.07
89	2,3-Dimethyl-5-ethylpyrazine	15707-34-3	Pyrazines	0.20 ± 0.00	ND	ND	ND
90	Pyrazine, tetramethyl-	1124-11-4	Pyrazines	1.47 ± 0.02	1.88 ± 0.05	1.41 ± 0.01	4.10 ± 0.01
91	Pyrazine, 2-ethenyl-6-methyl-	13925-9-2	Pyrazines	0.30 ± 0.08	ND	1.54 ± 0.02	4.51 ± 0.05
92	2,3-Dimethyl-5-isopentylpyrazine	18450-1-6	Pyrazines	0.14 ± 0.09	0.03 ± 0.01	0.10 ± 0.01	0.46 ± 0.09
93	Pyrazine, 2-ethyl-5-methyl-	13360-64-0	Pyrazines	ND	ND	3.51 ± 0.07	9.89 ± 0.01
94	Pyrazine, 2-(n-propyl)-	18138-3-9	Pyrazines	ND	ND	0.04 ± 0.00	0.13 ± 0.03
95	Pyrazine, 3,5-diethyl-2-methyl-	18138-5-1	Pyrazines	ND	0.02 ± 0.01	0.36 ± 0.04	1.15 ± 0.03
96	Pyrazine, 2-methyl-3-(2-propenyl)-	55138-62-0	Pyrazines	ND	ND	0.39 ± 0.04	1.51 ± 0.09
97	Pyrazine, (1-methylethenyl)-	38713-41-6	Pyrazines	ND	ND	0.17 ± 0.06	0.51 ± 0.05
98	Acetylpyrazine	22047-25-2	Pyrazines	ND	ND	0.84 ± 0.12	2.95 ± 0.04
99	Pyrazine, 2-methyl-5-(1-propenyl)-	18217-82-8	Pyrazines	ND	ND	0.08 ± 0.05	0.64 ± 0.08
100	Ethanone, 1-(3,5-dimethylpyrazinyl)-	54300-8-2	Pyrazines	ND	ND	0.26 ± 0.08	ND
101	Pyrazine, 2-ethenyl-5-methyl-	13925-8-1	Pyrazines	ND	ND	0.22 ± 0.03	0.87 ± 0.03
102	2-Butanone	78-93-3	Ketones	0.06 ± 0.03	0.08 ± 0.03	0.13 ± 0.02	0.59 ± 0.07
103	2,3-Butanedione	431-3-8	Ketones	0.16 ± 0.03	0.13 ± 0.06	0.30 ± 0.06	0.76 ± 0.09
104	2,3-Pentanedione	600-14-6	Ketones	0.03 ± 0.01	0.03 ± 0.02	0.25 ± 0.05	0.57 ± 0.06
105	2-Heptanone	110-43-0	Ketones	0.44 ± 0.01	0.43 ± 0.07	0.60 ± 0.08	1.68 ± 0.09
106	2-Heptanone, 3-methyl-	2371-19-9	Ketones	0.08 ± 0.01	ND	0.06 ± 0.00	ND
107	4-Heptanone, 2-methyl-	626-33-5	Ketones	0.10 ± 0.02	0.04 ± 0.00	0.06 ± 0.02	ND
108	2-Octanone	111-13-7	Ketones	0.07 ± 0.02	0.07 ± 0.03	0.14 ± 0.02	0.40 ± 0.05
109	5-Hepten-2-one, 6-methyl-	110-93-0	Ketones	0.18 ± 0.04	0.18 ± 0.11	0.24 ± 0.04	0.85 ± 0.07
110	2-Nonanone	821-55-6	Ketones	0.04 ± 0.01	0.08 ± 0.02	ND	ND
111	2-Cyclohexen-1-one	930-68-7	Ketones	0.76 ± 0.07	1.31 ± 0.08	0.91 ± 0.06	3.79 ± 0.03
112	2-Undecanone	112-12-9	Ketones	0.48 ± 0.05	0.77 ± 0.08	0.43 ± 0.08	2.49 ± 0.08
113	Cyclohexanone, 5-methyl-2-(1-methylethylidene)-	15932-80-6	Ketones	0.14 ± 0.01	ND	ND	ND
114	Bicyclo[3.1.1]hept-3-en-2-one, 4,6,6-trimethyl-	1196-1-6	Ketones	0.06 ± 0.01	0.09 ± 0.10	0.03 ± 0.01	0.24 ± 0.10
115	1,2-Cyclopentanedione, 3-methyl-	765-70-8	Ketones	0.12 ± 0.05	0.04 ± 0.01	0.34 ± 0.07	1.57 ± 0.06
116	5,9-Undecadien-2-one, 6,10-dimethyl-	3796-70-1	Ketones	0.14 ± 0.02	0.16 ± 0.01	ND	0.67 ± 0.06
117	trans-3-Nonen-2-one	18402-83-0	Ketones	ND	0.01 ± 0.00	0.05 ± 0.01	0.23 ± 0.08
118	1,4-Cyclohex-2-enedione	4505-38-8	Ketones	ND	ND	0.64 ± 0.08	2.74 ± 0.08
119	Cyclohexanone, 2-butyl-	1126-18-7	Ketones	ND	ND	0.06 ± 0.02	0.33 ± 0.08
120	2-Cyclopenten-1-one, 3-ethyl-2-hydroxy-	21835-1-8	Ketones	ND	ND	0.16 ± 0.01	0.75 ± 0.08
121	3-Penten-2-one	3102-33-8	Ketones	ND	ND	ND	0.75 ± 0.02
122	2,3-Octanedione	585-25-1	Ketones	ND	ND	ND	0.17 ± 0.02
123	Dodecane	112-40-3	Others	0.29 ± 0.07	0.69 ± 0.06	0.23 ± 0.02	1.69 ± 0.05
124	Furan, 2-pentyl-	3777-69-3	Others	0.47 ± 0.04	0.25 ± 0.06	0.99 ± 0.03	2.76 ± 0.03
125	Longifolene	475-20-7	Others	0.72 ± 0.01	1.65 ± 0.02	0.54 ± 0.01	3.96 ± 0.02
126	3(2H)-Furanone, dihydro-2-methyl-	3188-0-9	Others	0.03 ± 0.02	0.03 ± 0.02	0.03 ± 0.01	0.10 ± 0.02
127	Ethanone, 1-(2-pyridinyl)-	1122-62-9	Others	0.04 ± 0.01	0.03 ± 0.02	0.29 ± 0.06	1.30 ± 0.04
128	Ethanone, 1-(1H-pyrrol-2-yl)-	1072-83-9	Others	1.16 ± 0.02	1.27 ± 0.03	2.07 ± 0.08	8.40 ± 0.09
129	3-Furanmethanol	4412-91-3	Others	0.86 ± 0.02	0.55 ± 0.07	9.66 ± 0.05	33.34 ± 0.11
130	Phenol	108-95-2	Others	1.27 ± 0.04	ND	ND	ND
131	Eugenol	97-53-0	Others	0.02 ± 0.00	0.04 ± 0.01	0.02 ± 0.00	0.15 ± 0.00
132	1H-Pyrrole-2-carboxaldehyde, 1-methyl-	1192-58-1	Others	0.28 ± 0.07	0.07 ± 0.05	0.15 ± 0.02	0.72 ± 0.06
133	2-Furanmethanol, 5-methyl-	3857-25-8	Others	ND	ND	0.94 ± 0.02	3.70 ± 0.03
134	Furan, 3-methyl-	930-27-8	Others	ND	ND	0.02 ± 0.01	0.10 ± 0.03
135	Pyridine, 2-methyl-	109-6-8	Others	ND	ND	0.05 ± 0.02	0.13 ± 0.03
136	3-Furaldehyde	498-60-2	Others	ND	ND	0.83 ± 0.04	ND
137	Ethanone, 1-(2-furanyl)-	1192-62-7	Others	ND	ND	0.94 ± 0.04	3.67 ± 0.07
138	1,5-Dimethyl-2-pyrrolecarbonitrile	56341-36-7	Others	ND	ND	0.06 ± 0.02	0.28 ± 0.01
139	trans-Furfurylideneacetone	41438-24-8	Others	ND	ND	0.05 ± 0.01	0.25 ± 0.05
140	Quinoxaline, 2-methyl-	7251-61-8	Others	ND	ND	0.09 ± 0.01	0.13 ± 0.02
141	Furyl hydroxymethyl ketone	17678-19-2	Others	ND	0.09 ± 0.01	0.14 ± 0.01	1.27 ± 0.06
142	1H-Pyrrole-2-carboxaldehyde	1192-58-1	Others	ND	0.07 ± 0.05	0.15 ± 0.02	0.72 ± 0.06
143	2,5-Dimethylfuran-3,4(2H,5H)-dione	68755-49-7	Others	ND	ND	0.55 ± 0.03	3.01 ± 0.03
144	Phenol, 3-ethyl-	620-17-7	Others	ND	0.04 ± 0.02	0.06 ± 0.02	0.15 ± 0.02
145	Caryophyllene	87-44-5	Others	ND	0.08 ± 0.01	ND	0.54 ± 0.07
146	Naphthalene	91-20-3	Others	ND	0.05 ± 0.03	ND	ND
147	Phenol, 2-ethyl-	90-0-6	Others	ND	0.14 ± 0.09	ND	ND
148	p-Cresol	106-44-5	Others	ND	0.29 ± 0.01	ND	ND
149	Phenol, 3-methyl-	108-39-4	Others	ND	0.27 ± 0.09	0.17 ± 0.03	0.72 ± 0.05
150	trans-Isoeugenol	5932-68-3	Others	ND	0.03 ± 0.00	ND	ND

ND, not detected.
